# Salvianolic acid C alleviates acute kidney injury by restoring fructose-1,6-bisphosphatase 1-mediated gluconeogenesis

**DOI:** 10.1080/0886022X.2026.2629902

**Published:** 2026-03-08

**Authors:** Jianing Chen, Yujie Li, Peihui Zhou, Shanshan Zou, Jiaqi Zhao, Ming Wu, Li Wang

**Affiliations:** a Department of Nephrology, Shanghai Ninth People’s Hospital, Shanghai Jiao Tong University School of Medicine, Shanghai, P. R. China; b Key Laboratory of Liver and Kidney Diseases, Ministry of Education, Shanghai Key Laboratory of Traditional Chinese Clinical Medicine, Department of Nephrology, Shuguang Hospital Affiliated to Shanghai University of Traditional Chinese Medicine, TCM Institute of Kidney Disease of Shanghai University of Traditional Chinese Medicine, Shanghai, P. R. China

**Keywords:** Acute kidney injury, cisplatin, ischemia reperfusion injury, salvianolic acid C, gluconeogenesis, fructose-1, 6-bisphosphatase 1

## Abstract

This research was targeted to investigate the pharmacological effects and mechanisms of Salvianolic acid C (SAC), a natural compound extracted from the Chinese traditional herbal Danshen (*Salviae Miltiorrhizae Radix et Rhizoma*), in acute kidney injury (AKI). Male C57BL/6J mice were used to create animal models and SAC (10 mg/kg, i.p.) or saline was administered 24 h before modeling. In both ischemia reperfusion injury (IRI) induced AKI and cisplatin (Cis) induced AKI models, SAC significantly alleviated the decline of renal function and destruction of renal pathological structure, which were correlated with reduced expression of renal injury markers and inhibited renal cell apoptosis. Non-targeted metabolomics sequencing revealed that the gluconeogenesis pathway was downstream of the renal protective effect of SAC in AKI. Further experiments revealed that the expression of key gluconeogenic enzyme fructose-1,6-bisphosphatase 1 (FBP1) was significantly decreased in AKI kidneys. AKI induced the reduction in glucose levels from serum and increase in lactate levels from serum and renal tissue. All of these metabolic changes were improved after the SAC treatment. The protective effects of SAC were reversed by an FBP1 inhibitor. Peroxisome proliferator-activated receptor gamma coactivator 1-alpha (PGC1α) and forkhead box O1 (FOXO1), identified as gluconeogenesis promoting factor, were decreased after injection of cisplatin, which were reversed by SAC. Finally, molecular docking and SPR experiments revealed the direction interaction between FBP1 and SAC. Collectively, SAC possibly exerts its effect through a dual regulatory mechanism: by modulating the FOXO1/PGC1α/FBP1 signaling axis and *via* direct interaction with FBP1.

## HIGHLIGHTS

- SAC alleviates both IRI and Cisplatin induced AKI.
- SAC improves cisplatin-induced AKI through FBP1-mediated gluconeogenesis.
- SAC modulates FBP1-mediated gluconeogenesis through a dual regulatory mechanism.

## Introduction

Acute kidney injury (AKI), a critical clinical syndrome characterized by rapid decline in glomerular filtration rate, poses a substantial burden characterized by persistently high morbidity and mortality. Beyond the immediate clinical crisis, AKI is now recognized as a pivotal event that alters long-term outcomes; despite supportive care advances, it increases the risk of premature death, chronic kidney disease, and progression to kidney failure compared with the general population [1,2]. The pathological hallmarks of AKI involve tubular epithelial cell necrosis, mitochondrial dysfunction, and dysregulated inflammatory-oxidative cascades [3–5]. Etiologically, AKI can be classified into prerenal (hypovolemia, reduced cardiac output), intrinsic renal (ischemia, nephrotoxic agents, sepsis), and postrenal (urinary tract obstruction) categories, with intrinsic renal AKI accounting for over 50% of cases [6], predominantly caused by ischemic injury or drug-induced nephrotoxicity [7,8], with cisplatin (cis-diamminedichloroplatinum II, Cis) being the most prevalent causative agent. Recent research has further elucidated the intricate roles of immune cells, such as lymphocytes and innate immune cells and novel forms of regulated cell death in AKI [9]. Notably, tubular injury biomarkers such as kidney injury molecule-1 (KIM-1) and neutrophil gelatinase-associated lipocalin (NGAL) exhibit high diagnostic sensitivity and specificity for early AKI detection, correlating strongly with the severity of tubulointerstitial inflammation [10,11]. Although certain pharmacological interventions may delay disease progression, renal replacement therapy remains the sole definitive treatment for end-stage renal disease [12]. Recent multidisciplinary efforts, such as the Kidney Precision Medicine Project, have begun to identify molecular subtypes of human AKI, highlighting the need for targeted therapies that address specific pathophysiological pathways [13].

Emerging evidence highlights the pivotal role of renal gluconeogenesis in metabolic regulation, with the kidney contributing up to 40% of systemic glucose production during fasting or stress conditions [14]. For instance, damage to peritubular capillaries—a key process in AKI—implicates factors like vascular endothelial growth factor A, which exhibits dual roles in acute injury *versus* fibrosis phases [15]. Additionally, neuropeptide Y has been identified as a protective agent in AKI, modulating macrophage activation and inflammation [16]. Notably, it has been revealed that the expression levels of the rate-limiting enzymes for gluconeogenesis, including pyruvate carboxylase, phosphoenolpyruvate carboxykinase 1 (PCK1), fructose-1,6-bisphosphatase 1 (FBP1), and glucose-6-phosphatase catalytic subunit (G6PC) are progressively downregulated during chronic kidney disease (CKD) progression [17]. Legouis et al. revealed that impaired renal gluconeogenesis during ischemia-reperfusion injury (IRI)-induced AKI triggers systemic metabolic alterations [18]. However, the precise mechanisms connecting gluconeogenic dysfunction to AKI pathogenesis remain poorly defined.

Peroxisome proliferator-activated receptor γ coactivator 1α (PGC1α) and its partner transcriptional factor, forkhead box O1 (FOXO1), play significant roles in kidney diseases [19,20]. Downregulation of PGC1α is strongly associated with the progression of AKI [20]. PGC1α is a crucial regulator of mitochondrial biogenesis and energy metabolism, including carbohydrate metabolism and lipid metabolism [21]. As an upstream transcription factor, FOXO1 can directly bind to the promoters of gluconeogenesis-related genes such as PCK1 and G6PC, thereby promoting their expression [22]. Additionally, FOXO1 can interact with PGC-1α to promote the expression of gluconeogenesis genes [23]. Activation of the FOXO1/PGC1α signaling pathway can alleviate renal injury [19] and promote disease recovery, however whether this protection is linked to activation of renal gluconeogenesis is not known.

Salvianolic acid C (SAC), a natural compound from Danshen, has shown renoprotective effects in chronic kidney disease models by mitigating oxidative stress and inflammation [24,25]. Specifically, SAC improves renal function by modulating oxidative stress and inflammatory pathways [26,27]. However, its efficacy in AKI, especially its potential to modulate gluconeogenesis, is unexplored.

In this study, we aimed to evaluate the effect of SAC in AKI and elucidate its underlying mechanisms. Using murine models of IRI and cisplatin-induced AKI, we assessed SAC’s efficacy in preserving renal function and histopathological integrity. Furthermore, integrated untargeted metabolomics and transcriptomics analyses were employed to identify SAC-regulated metabolic pathways.

## Methods

### Animal models

Male C57BL/6J mice (6–8-week-old, body weight 22 ± 2 g) were utilized. Animals were randomly assigned to experimental groups using a computer-generated random number table, with stratification by body weight to ensure homogeneity across groups. The randomization was performed by an investigator not involved in subsequent procedures to minimize bias. All mice were anesthetized by intraperitoneal injection of a combination of Zoletil 50 (Cat# zoletil50, Hangzhou Shuo Ye, Hangzhou, China) (50 mg/kg) and dexmedetomidine hydrochloride Injection (Cat# 2011, Hangzhou Shuo Ye, Hangzhou, China) (0.5 mg/kg). IRI-AKI: For establishing the IRI-AKI model, animals underwent anesthesia, bilateral renal arterial clamping for 30 min, and subsequent reperfusion over 24 h. Briefly, the abdominal cavity was opened, intestines were exteriorized, and renal arteries were clamped with non-traumatic vascular clamps for 30 min. SAC (10 mg/kg, i.p.) or saline was administered 24 h pre-surgery. The SAC dose of 10 mg/kg was selected based on its established efficacy and safety profile in prior renal injury studies [25]. Mice were randomized into four experimental groups: Sham + vehicle, Sham + SAC, IRI + vehicle, and IRI + SAC (*n* = 6/group). Mice were sacrificed for sampling after 30 min/24 h ischemia/reperfusion. Cisplatin-induced AKI: Mice received cisplatin (24 mg/kg, i.p.) dissolved in saline (1 mg/mL, 37 °C water bath with agitation). SAC (10 mg/kg) or vehicle (saline with 10% DMSO) was injected 24 h pre-cisplatin. Mice were randomly divided into Saline + vehicle, Saline + SAC, Cis + vehicle, and Cis + SAC (*n* = 6/group). Salvianolic acid C (Cat# T3149, TargetMol, Shanghai, China) was dissolved in DMSO (50 mg/mL stock). After dilution in saline to 5 mg/mL, intraperitoneal injection was performed. All mice were sacrificed for sampling 72 h after cisplatin injection. Additionally, in FBP1 inhibition experiments, another individual group is set as Cis + SAC + FBP1 inhibitor, in which the FBPase-1 inhibitor, Benzoxazole benzenesulfonamides (Cat# T22081, TargetMol, TargetMol, Shanghai, China), was administered at 5 mg/kg, i.p., 24 h before cisplatin administration. The inhibitor was dissolved in DMSO as a 50 mg/mL stock solution and diluted in normal saline to a working concentration of 1 mg/mL. This inhibitor is a potent allosteric inhibitor of fructose-1,6-bisphosphatase (FBPase-1) with high selectivity (IC_50_ = 0.57 μM), as characterized in previous structural studies [28].

### Cell culture and intervention

Human kidney proximal tubular epithelial cells (HK2 cells) were acquired from the Shanghai Institute of Biological Sciences Cell Bank (Chinese Academy of Sciences, Shanghai, China). Cells were maintained in Dulbecco’s Modified Eagle Medium/Nutrient Mixture F-12 (DMEM/F12) supplemented with 10% fetal bovine serum (FBS) and 0.5% penicillin/streptomycin solution. All cultures were incubated at 37 °C in a humidified atmosphere containing 5% CO_2_. For SAC dose-dependent in cisplatin model, HK2 cells were seeded in six-well plates at an appropriate density. Upon reaching ∼50% confluence, cells were synchronized by serum starvation using medium containing 0.5% FBS for 8 h. Following synchronization, cells were exposed to cisplatin (10 μM) to establish an *in vitro* injury model. Concurrently, SAC was administered at three different concentrations (10, 30, and 100 μM) with cisplatin. After 24 h of continuous exposure, both RNA and total protein were harvested for subsequent molecular analyses.

For gene knockdown experiments, HK2 cells were transfected with FBP1-specific siRNA or non-targeting control siRNA using Lipofectamine™ 3000 (CAT#L3000001, Thermo Fisher Scientific, Carlsbad, California, USA) reagent according to manufacturer’s instructions. FBP1-targeting siRNA (si-FBP1) was designed and synthesized by GenePharma Co., Ltd. (Shanghai, China), and the specific sequence used in this study was as follows: si-FBP1-human-799: Sense strand: 5′-GGACAAGGAUGUGAAGAUATT-3′, Antisense strand: 5′-UAUCUUCACAUCCUUGUCCTT-3′. A non-targeting scrambled siRNA (si-NC) was used as a negative control:si-NC:Sense strand: 5′-UUCUCCGAACGUGUCACGUTT-3′, Antisense strand:5′-ACGUGACACGUUCGGAGAATT-3′. Briefly, cells were plated in antibiotic-free medium 24 h before transfection to achieve 60–70% confluence at the time of transfection. The siRNA-Lipofectamine 3000 complexes were prepared in serum-free medium and added to cells for 6 h, after which the transfection mixture was replaced with complete growth medium. Cells were incubated for an additional 18 h to achieve a total transfection duration of 24 h. The experimental groups included: (1) Control, (2) Cisplatin (10 μM), (3) Cisplatin + SAC (30 μM), and (4) Cisplatin + FBP1 siRNA. Following transfection, cells were treated according to the designated groups for 24 h before sample collection.

### Renal function measurements

The blood was collected *via* intracardiac puncture under anesthesia, centrifuged at 3,000 × g for 15 min, and the supernatant (serum) was aliquoted and stored at −80 °C for subsequent analysis of serum creatinine (Scr) and blood urea nitrogen (BUN). Scr and BUN levels were detected utilizing commercial kits (Scr: Cat# C011-2-1, Nanjing Jiancheng, Nanjing, Jiangsu, China; BUN: Cat# C013-2-1, Nanjing Jiancheng, Nanjing, Jiangsu, China) following the protocols provided by the manufacturer.

### RNA extraction and real-time fluorescence quantitative PCR

Total RNA was isolated from mouse kidney tissues or HK2 cells using an RNA Rapid Extraction Kit (Cat# RN001, ESscience, Shanghai, China) according to the manufacturer’s protocol. qPCR amplification was conducted on a Real-Time PCR System with SYBR Green premix (Cat# RR 420 A, Takara Bio, Shiga, Japan) according to standard protocols. For quantitative analysis, TB Green Premix Ex Taq (Cat# RR820A, Takara Bio, Shiga, Japan) was employed as the detection reagent. The 20 μL reaction system contained 10 μL 2× Premix, 0.8 μM forward/reverse primers (Table 1), and 2 μL diluted cDNA (5×). Thermal cycling parameters included: 95 °C for 30 s; 35 cycles of 95 °C for 5 s and 60 °C for 30 s; followed by melt curve analysis (65–95 °C, 0.5 °C/s) [29]. All samples were run in triplicate with Gapdh as the endogenous control. The primer sequences are listed in Table 1.

**Table 1.** Sequences of rt-qPCR primers.

Gene name	Primer sequence
Mouse-Gapdh	Forward, 5′-AGGTCGGTGTGAACGGATTTG-3′
	Reverse, 5′-TGTAGACCATGTAGTTGAGGTCA-3′
Mouse-Fbp1	Forward, 5′-GCTCTGCACCGCGATCA-3′
	Reverse, 5′-ACATTGGTTGAGCCAGCGATA-3′
Mouse-Pck1	Forward, 5′-GGTTCCCAGGGTGCATGAAA-3′
	Reverse, 5′-CACGTAGGGTGAATCCGTCAG-3′
Mouse-G6pC	Forward, 5′-CCTCAGGAATGCCTTCTACG-3′
	Reverse, 5′-TCTCCAATCACAGCTACCCA-3′
Human-βactin	Forward, 5′-ATTGCCGACAGGATGCAGAA-3′
	Reverse, 5′-CGGACTCGTCATACTCCTGC-3′
Human-Fbp1	Forward, 5′-CTTTGCCACGTGTGTTCTCG-3′
	Reverse, 5′-ACGGACACAAGGCAATCGAT-3′
Human-G6pc	Forward, 5′-GGCTCTCAACTCCAGCATGT-3′
	Reverse, 5′-AGGACGAGGGAGGCTACAAT-3′
HumanPckl	Forward, 5′-CTGAACCTCTCGGCCAAAGT-3′
	Reverse, 5′-AGGATGCCCTCTTCCTCCAT-3′
Human-Kim-1	Forward, 5′-ATTGTTGCCGTGTTGAGCAC-3′
	Reverse, 5′-GTAGTCGTGACCTTGGGTGG-3′
Human-Ngal	Forward, 5′-GTATGTGGTAGGCCTGGCAG-3′
	Reverse, 5′-CAGGACGGAGGTGACATTGT-3′

### Western blotting

Renal tissues or HK2 cells were homogenized in ice-cold RIPA lysis buffer (Cat# P0013B, Beyotime Biotechnology, Shanghai, China) containing 1 mM PMSF (Cat# ST505, Beyotime Biotechnology, Shanghai, China) using a cryogenic grinder (65 Hz, 3 cycles of 60 s grinding/2 s pause). Lysates were centrifuged at 12,000 ×*g* (4 °C, 10 min), and supernatants were quantified *via* BCA protein assay (Cat# WB6501, New Cell & Molecular Biotech, Suzhou, Jiangsu, China) with a standard curve (0.0625–2 μg/μL). Protein samples (20 μg/lane) mixed with 5 × SDS loading buffer (Cat# WB2001, New Cell & Molecular Biotech, Suzhou, Jiangsu, China) were denatured (95 °C, 5 min), separated on 8% SurePAGE™ precast gels (Cat# M00657, GenScript, Nanjing, Jiangsu, China), and transferred to PVDF membranes (Cat# L00733C/L00734C, GenScript, Nanjing, Jiangsu, China) using a semi-dry transfer system (Standard mode). Membranes were blocked with 5% skim milk in TBST (Cat# C520009, Sangon Biotech, Shanghai, China) for 1 h, then incubated overnight (4 °C) with primary antibodies (Table 2). After TBST washing, membranes were exposed to HRP-secondary antibodies for 60 min at room temperature. Signals were detected with ECL reagent (Cat# WBKLS0500, Merck Millipore, Burlington, MA, USA) and quantified *via* ImageJ software (Media Cybernetics, Rockville, MD) as detailed in Supplementary Tables B2–B13.

**Table 2.** Primary antibody information for Western blot.

Antibody	Catalog number and company	Dilution ratio
β-actin	Cat# AC038, ABclonal, Wuhan, China	1:50,000
NGAL	Cat# A2092, ABclonal, Wuhan, China	1:500
KIM-1	Cat# A2831, ABclonal, Wuhan, China	1:1000
Bax	Cat# #2772, Cell Signaling Technology, Danvers, USA	1:1000
Bcl-2	Cat# #15071, Cell Signaling Technology, Danvers, USA	1:1000
FBP1	Cat# A11664, ABclonal, Wuhan, China	1:1000
PCK1	Cat# A22172, ABclonal, Wuhan, China	1:2000
G6PC	Cat# A21168, ABclonal, Wuhan, China	1:2000

### Gluconeogenic intermediate measurement

Serum was collected from mice. Proteins were extracted from the renal cortex by mechanical disruption using normal saline. The lactate concentration of serum and renal tissue was assessed using a lactate assay kit (Cat# A019-2-1, Nanjing Jiancheng, Nanjing, Jiangsu, China), with the OD value measured at 530 nm. All procedures followed the manufacturer’s guidelines and were normalized to protein concentration.

### Glucose concentration measurement

The glucose levels of serum and renal tissue were measured using a Glucose Assay Kit (Cat# A154-1-1, Nanjing Jiancheng, Nanjing, Jiangsu, China). Absorbance was measured at 505 nm with a microplate reader, and glucose concentrations were calculated based on the OD value of the standard sample. All procedures followed the manufacturer’s guidelines.

### Hematoxylin-eosin (HE) staining and tubular injury scoring

Renal tissues underwent fixation with 4% paraformaldehyde followed by paraffin embedding. Consecutive sections (4–5 μm thickness) from paraffin blocks were subjected to HE staining according to the Hematoxylin-Eosin Staining Kit (Cat# G1076, Beijing, China) manufacturer’s protocol. Deparaffinized sections were treated with hematoxylin (3 min), differentiated in 1% acid ethanol, and counterstained with eosin (15 s). Morphological evaluation was performed using a NIKON ECLIPSE E100 upright microscope (Nikon, Tokyo, Japan) at 200× magnification. Tubular injury in the S3 segment was blindly scored across 10 random fields per section: 1: <10% (minor vacuolation); 2: 10–25% (focal dilation); 3: 25–50% (basement membrane rupture); 4: 50–75% (extensive casts); 5: >75% (necrosis/fibrosis).

### Periodic acid-Schiff (PAS) staining

PAS staining was performed with a commercial kit (Cat# G1008, Servicebio, Wuhan, Hubei, China). Sections were oxidized with 0.5% periodic acid (10 min, RT), incubated with Schiff reagent (15 min, 37 °C), and counterstained with hematoxylin. PAS-positive glycoproteins (magenta) were visualized using the same microscope. The PAS-stained positive area was quantified through ImageJ software.

### TUNEL staining

Kidney paraffin sections (5 µm) were deparaffinized, antigen-retrieved with proteinase K (Cat# G1205, Servicebio, Wuhan, Hubei, China), and incubated with TUNEL reaction mixture (TMR Red, TUNEL Kit, Cat# G1502, Servicebio, Wuhan, Hubei, China) at 37 °C for 1 h. DAPI was used to counterstain Nuclei. Sections were mounted with anti-fade medium and imaged using a fluorescence microscope (Nikon Eclipse C1, Nikon, Tokyo, Japan). TUNEL-positive cells (red, excitation/emission: 510–561/590 nm) and DAPI (blue, 330–380/420 nm) were quantified using ImageJ software. Note that although the TUNEL-positive cells were originally stained red, they were displayed in green for better visualization after scanning.

### Immunohistochemistry (IHC)

Kidney tissues were fixed in 4% paraformaldehyde, dehydrated, and cut into 5 µm thick sections. Paraffin sections were deparaffinized to water, antigenically repaired, endogenous peroxidase blocked, BSA blocked, primary antibody overnight at 4 °C, secondary antibody incubated, DAB developed, hematoxylin re-stained, blocked, and images were acquired. Primary antibodies used were Cleaved-caspase-3 (Cat# 9661, Cell Signaling Technology, Danvers, MA, USA), FOXO1 (Cat# GB12286, Servicebio, Wuhan, China), and PGC1α (Cat# 66369-1-IG, Servicebio, Wuhan, Hubei, China). The percentage of positive area in each sample was quantified using ImageJ software [30].

### Untargeted metabolomics sequencing

Untargeted metabolomics sequencing was performed using ultra-high performance liquid chromatography coupled with high-resolution mass spectrometry (UHPLC-Q Ex active HF-X, Thermo Fisher Scientific, Waltham, MA, USA). Renal tissue metabolites were systematically compared between cisplatin vehicle-treated (Cis group) and cisplatin plus SAC-treated (Cis + SAC group) mice. Chromatographic separation was achieved on an HSS T3 column (100 × 2.1 mm, 1.8 μm) with mobile phases consisting of 0.1% formic acid in 95% water/5% acetonitrile (A) and 0.1% formic acid in 47.5% acetonitrile/47.5% isopropanol/5% water (B). Mass spectrometry analysis was conducted in both positive and negative ionization modes (scan range: m/z 70–1050). Raw data were processed using the bioinformatics platform (Majorbio Cloud Platform, Shanghai, China) [31] for peak alignment, metabolite annotation, and differential metabolite screening (VIP >1.0, *p* < 0.05).

### Fluorescent homologous double labeling staining

Five-micrometer-thick renal sections mounted in paraffin underwent sequential deparaffinization and rehydration before microwave-assisted antigen retrieval. Before immunostaining, endogenous peroxidases were quenched with 3% hydrogen peroxide, and nonspecific binding sites were blocked with 3% bovine serum albumin. Sections were sequentially incubated with primary antibodies: LRP2 (Cat# GB112109, Servicebio, Wuhan, Hubei, China), a marker localized to renal tubules, and FBP1 (Cat# 12842-1-AP, Proteintech, Rosemont, IL, USA), overnight at 4 °C. After PBS washes, tissues were labeled with HRP-conjugated secondary antibodies. Tyramide signal amplification (TSA) was performed using fluorophores: iF488-Tyramide (Cat# G1231, Servicebio, Wuhan, Hubei, China), CY3-Tyramide (Cat# G1223, Servicebio, Wuhan, Hubei, China), and FITC-Tyramide (Cat# G1202, Servicebio, Wuhan, Hubei, China). After secondary antigen retrieval, nuclei were counterstained with DAPI, and autofluorescence was quenched. Slides were mounted and imaged under specific fluorescence channels: DAPI: Excitation 330–380 nm/Emission 420 nm (blue nuclei), 488 nm (FITC/iF488): Excitation 465–495 nm/Emission 515–555 nm (green), and CY3: Excitation 510–560 nm/Emission 590 nm (red).

### Molecular docking

The protein FBP1 (UniProt ID: P09467) was retrieved from UniProt and preprocessed by removing water molecules, adding hydrogens, and assigning charges. The small molecule Salvianolic acid C (PubChem CID: 13991590) was obtained from PubChem, energy-minimized, and analyzed for rotatable bonds. Molecular docking was performed using AutoDock 4.2 with the Lamarckian Genetic Algorithm under semi-flexible conditions. The lowest-energy conformation from the docking results was selected for subsequent visualization and analysis.

### Surface-plasmon resonance (SPR)

SPR was conducted on a BIAcore 1K with CM5 chips at 25 °C. CM5 sensor surfaces were activated with EDC/NHS (200 μM/50 μM, 10 μL/min, 420 s), followed by immobilization of protein (50 μg in 10 mM sodium acetate, pH 5.0; 10 μL/min, 420 s, duplicate runs). Reference channels were activated/blocked identically but immobilized with PBS (pH 5.0). After equilibration with PBS, serially diluted analytes (in PBS) were injected (10 μL/min, 150 s). Regeneration used 10 mM glycine-HCl (pH 2.0; 10 μL/min, 5 min). Data from sample channels (subtracted by reference) were analyzed *via* Biacore 1K Evaluation Software (1:1 Langmuir model) and visualized in Origin 7.

### Statistical analysis

Data were presented as mean ± standard deviation (X ± SD). Two-group comparisons were performed using *t*-tests, while comparisons among multiple groups were conducted using ANOVA. GraphPad Prism10.0 software (GraphPad Software, La Jolla, CA) was used for data analysis.

## Results

### SAC alleviates renal damages in IRI-AKI

**Figure 1.**
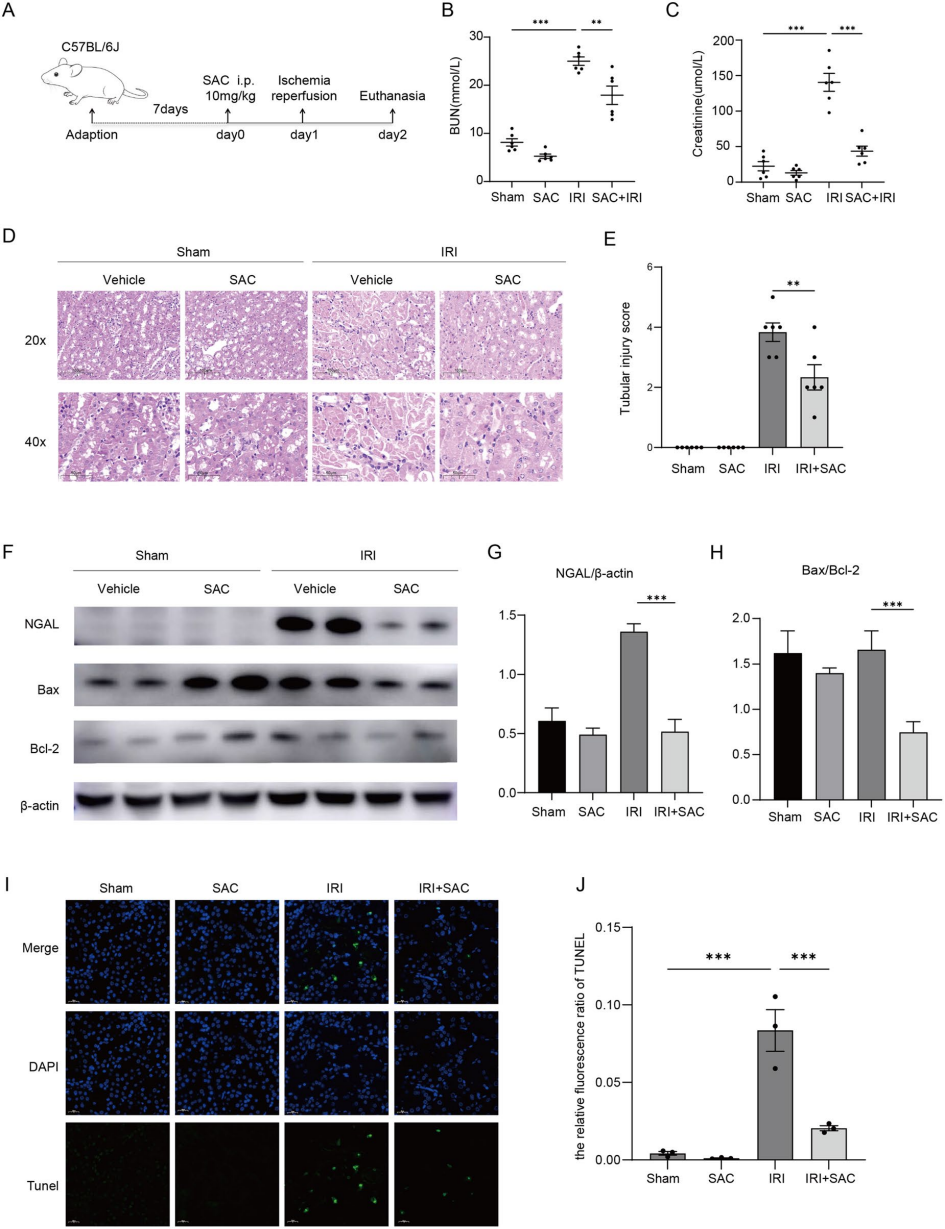
SAC attenuates renal IRI. (A) A schematic diagram illustrating the IRI-induced AKI model and the dosing schedule of the SAC treatment group (6 mice per group). (B, C) Serum creatinine (Scr) levels and blood urea nitrogen (BUN) levels. (D) Representative photomicrographs of H&E staining in renal tissues (original magnification ×20, ×40; Scale bar: 100 μm, 50 μm). (E) Quantitative assessment of tubular injury. (F) Representative Western blot images showing protein expression levels of NGAL, Bax, and Bcl-2. (G,H). Quantitative analysis of the Western blot results. The bar graphs represent the relative protein levels of NGAL, Bax, and Bcl-2, calculated as the ratio of the integrated density (IntDen) of each target band to that of the corresponding β-actin band. (I,J) Representative fluorescence micrographs and quantification of TUNEL staining in renal tissues (DAPI: blue, TUNEL: green). NS represents not significant. **p < 0.01. ***p < 0.001.

To evaluate the potential therapeutic efficacy of SAC in AKI, a mouse model of IRI was established (Figure 1(A)). Serum creatinine and blood urea nitrogen levels were significantly elevated in the IRI-Vehicle group, while these parameters were markedly attenuated after SAC treatment (Figures 1(B,C)). Histopathological examination of renal tissues through HE staining revealed pathological changes in the IRI-Vehicle group, including tubular epithelial cell swelling, tubular dilatation, cast formation, and nuclear dissolution. In contrast, mice treated with SAC exhibited ameliorated renal parenchymal damage and reduced tubular injury scores compared to the IRI-Vehicle group (Figures 1(D,E)). Furthermore, in IRI kidneys, SAC treatment reduced Ngal protein expression (Figures 1(F,G)). SAC induced upregulation of Bax and downregulation of Bcl-2, leading to a reduced Bax/Bcl-2 ratio in IRI kidneys (Figures 1(F–H)). TUNEL staining demonstrated a significant increase in apoptotic cells in renal tissues following IRI surgery, whereas SAC treatment effectively reduced the number of TUNEL-positive cells, supporting its anti-apoptotic properties in IRI kidneys (Figures 1(I,J)). Taken together, these data demonstrate that SAC exerts protective effects against IRI-induced renal injury in mice.

### SAC alleviates renal damage in cisplatin-induced AKI

**Figure 2.**
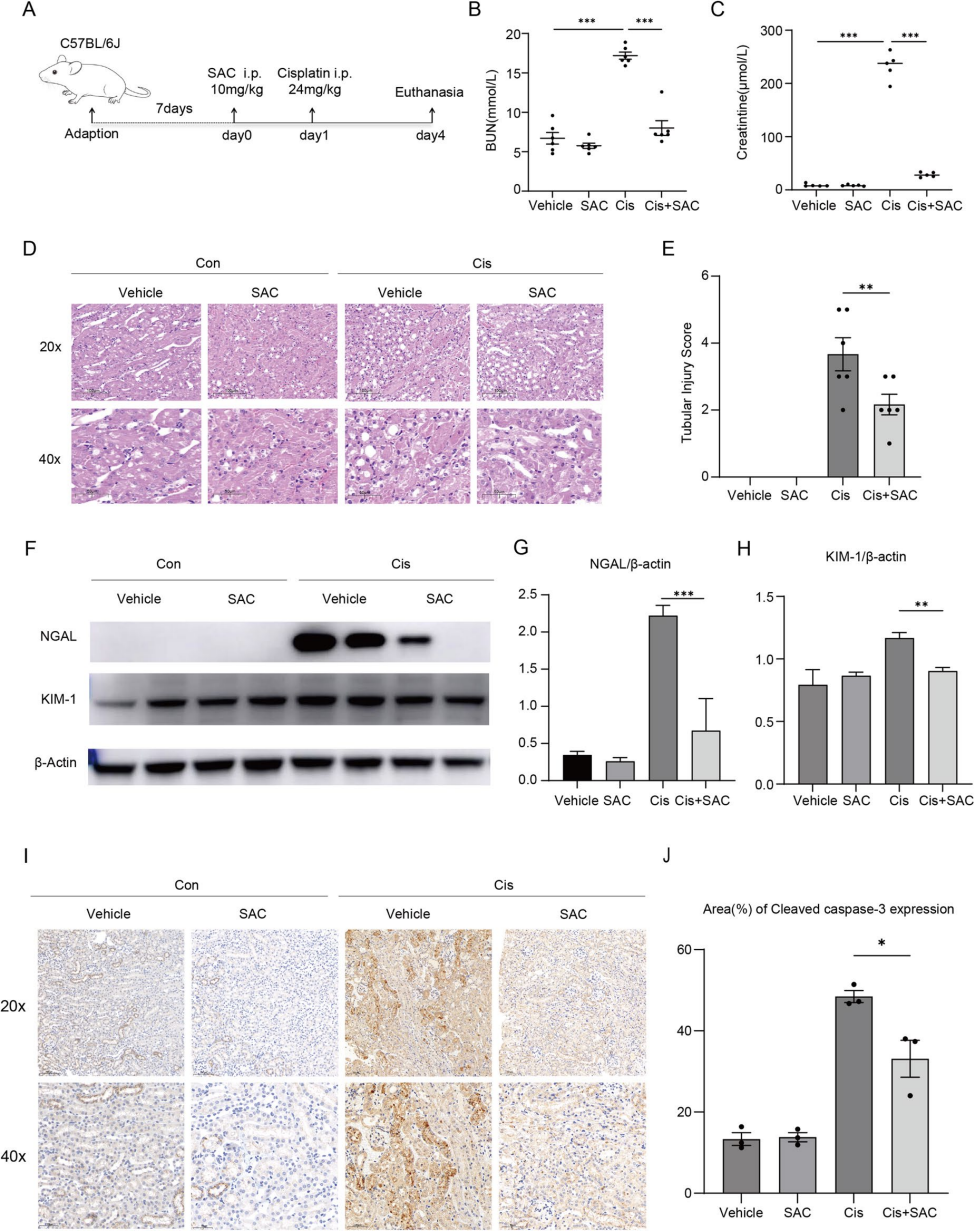
SAC provides renal protection against cisplatin-induced AKI. (A) A schematic diagram illustrating the cisplatin induced AKI model and the dosing schedule of the SAC treatment group (6 mice per group). (B,C) Alterations in Scr levels and BUN levels across experimental groups. (D) Representative photomicrographs of H&E staining in renal tissues (original magnification ×20, ×40; Scale bar: 100 μm, 50 μm). (E) Quantitative assessment of tubular injury. (F) Representative Western blot images showing protein expression levels of NGAL and KIM-1. (G,H). Quantitative analysis of the Western blot results. The bar graphs represent the relative protein levels of NGAL and KIM-1, calculated as the ratio of the integrated density (IntDen) of each target band to that of the corresponding β-actin band. Data are presented as mean ± SEM. (I,J) Representative photomicrographs and quantification of Cleaved-caspased-3 staining in renal tissues. NS represents not significant. *p < 0.05. **p < 0.01. ***p < 0.001.

A cisplatin induced AKI model was established to further confirm the effect of SAC in AKI (Figure 2(A)). SAC pretreatment significantly prevented the elevation of Scr and BUN levels in cisplatin mice (Figures 2(B,C)). Histopathological examination revealed an improved renal parenchymal structure in SAC-treated cisplatin mice, characterized by attenuated tubular epithelial cell swelling, decreased tubular dilatation, and diminished cast formation compared to the cisplatin group (Figures 2(D,E)). Furthermore, Western blotting analysis demonstrated that SAC pretreatment suppressed the upregulation of renal KIM-1 and NGAL induced by cisplatin (Figures 2(F–H)). Consistently, immunohistochemical staining further showed that SAC administration reversed cisplatin-triggered elevation of cleaved caspase-3, a marker of apoptosis, expression in renal tissues (Figures 2(I,J)). Collectively, these findings demonstrate that SAC confers protection against cisplatin-induced nephrotoxicity.

### Gluconeogenesis is a mechanism underlying SAC-mediated protection against AKI

**Figure 3.**
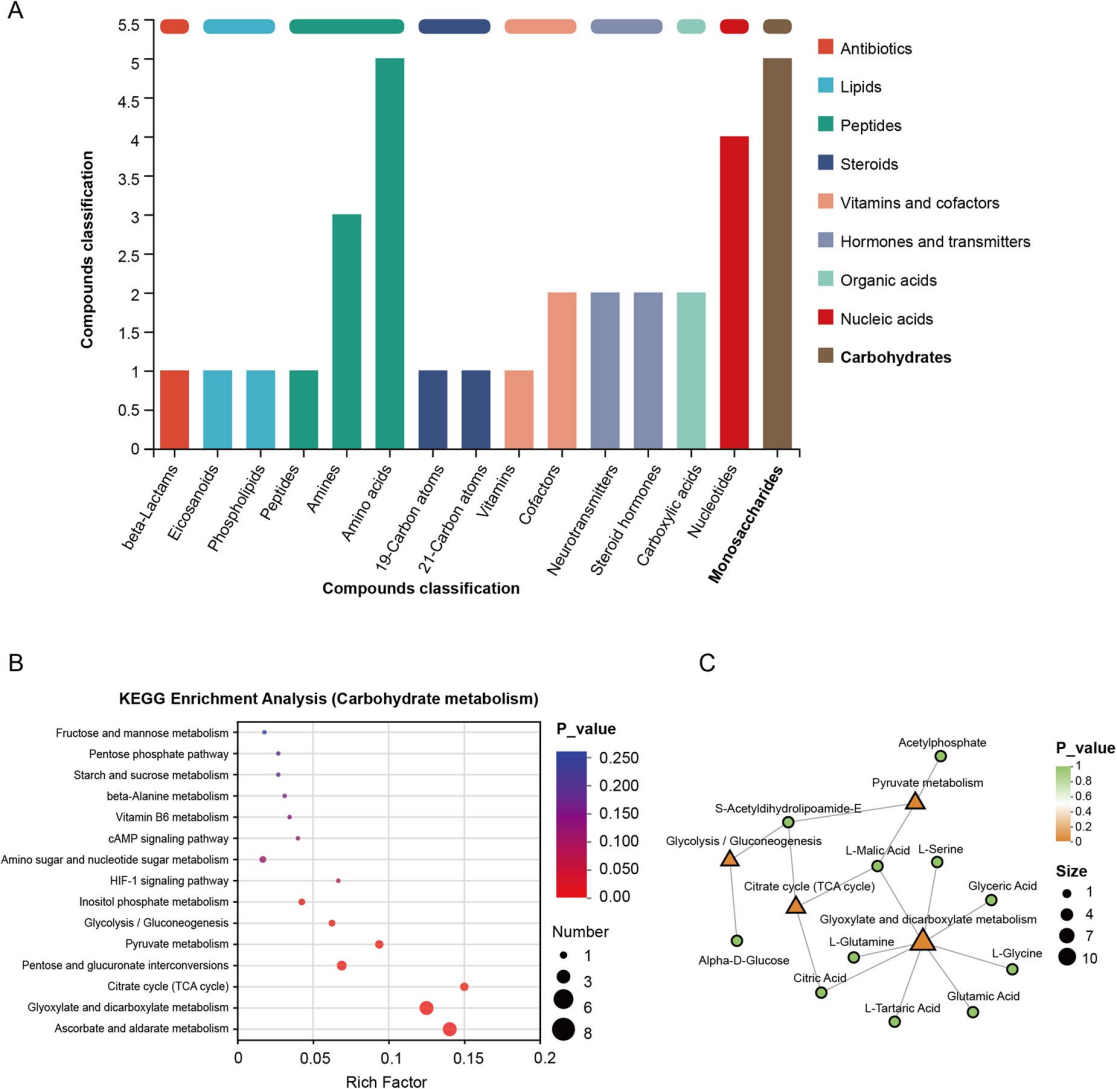
Untargeted metabolomic profiling of cisplatin-induced AKI mouse kidneys with or without SAC preconditioning. Six renal samples per group were collected for untargeted metabolomic profiling from mice upon cisplatin injection with or without SAC treatment. (A) Bar chart of KEGG compound classification statistics between Cis + Vehicle and Cis + SAC groups. (B) KEGG enrichment analysis focused on carbohydrate metabolism between Cis + Vehicle and Cis + SAC groups. (C) KEGG pathway enrichment analysis network diagram including glyoxylate and dicarboxylate metabolism, citrate cycle (TCA cycle), pyruvate metabolism, and glycolysis/gluconeogenesis.

Next, we explored the mechanism downstream of the effect of SAC on AKI by performing untargeted metabolomics analysis of cisplatin kidneys with or without SAC preconditioning. KEGG analysis revealed that carbohydrates and amino acids constituted the predominant classes of metabolites, followed by nucleotides and amines (Figure 3(A)), implicating glucose metabolism reprogramming as an important mechanism underlying SAC-mediated renoprotection in AKI.

Further KEGG analysis on carbohydrate metabolism demonstrated that glyoxylate and dicarboxylate metabolism (Rich factor = 0.125, *p* < 0.001), citrate cycle (TCA cycle) (Rich factor = 0.15, *p* < 0.001), and pyruvate metabolism (Rich factor = 0.093, *p* < 0.001) exhibited prominent enrichment characteristics (Figure 3(B)). Although the gluconeogenesis pathway itself showed moderate enrichment significance (Rich factor = 0.031, *p* = 0.0211), network topology analysis revealed extensive meta­bolite cross-talk between TCA cycle, glyoxylate/dicarboxylate metabolism, pyruvate metabolism, and glycolysis/gluconeogenesis pathways, implying a novel mechanistic paradigm wherein SAC modulates gluconeogenic flux to exert renal protective effects on AKI (Figure 3(C)).

### SAC restores gluconeogenesis in AKI

**Figure 4.**
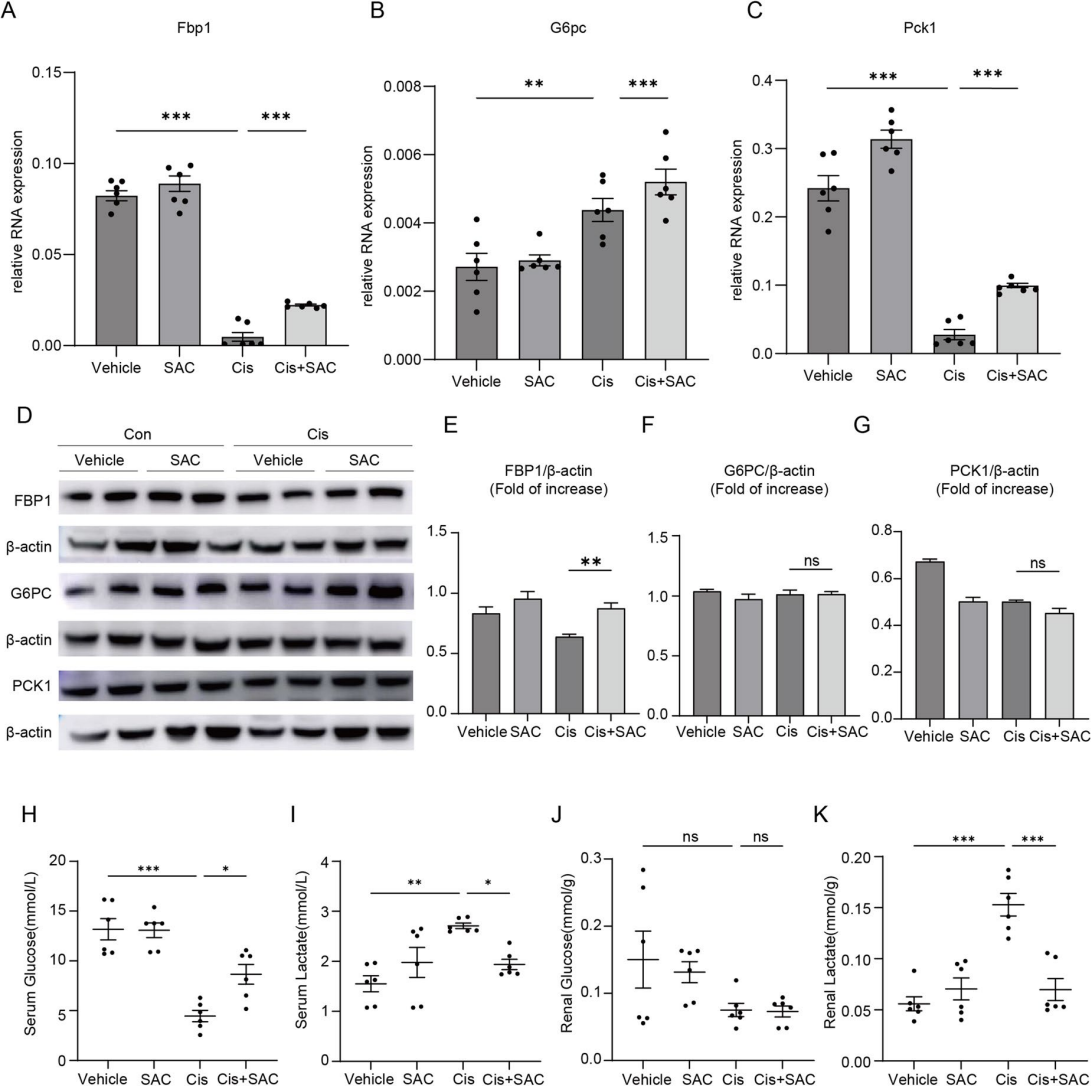
SAC promotes renal gluconeogenesis in cisplatin-induced AKI. (A–C) qPCR analysis of *Fbp1*, *G6pc*, and *Pck1*. (D) Representative Western blot images showing protein expression levels of FBP1, G6PC, and PCK1. (E–G) Quantitative analysis of the Western blot results. The bar graphs represent the relative protein levels of FBP1, G6PC, and PCK1, calculated as the ratio of the integrated density (IntDen) of each target band to that of the corresponding β-actin band. Data are presented as mean ± SEM. (H,I) Serum glucose and lactate assessment of mice in Vehicle, SAC, Cis and Cis + SAC groups. (J,K) Renal glucose and lactate assessment of mice in Vehicle, SAC, Cis, and Cis + SAC groups. NS represents not significant. *p < 0.05. **p < 0.01. ***p < 0.001.

To determine the role of gluconeogenesis in SAC-mediated protection against AKI, we examined renal expression of key rate-limiting enzymes of gluconeogenesis, including FBP1, G6PC, and PCK1. qPCR analysis revealed that renal expression of *Fbp1* and *Pck1* is reduced in cisplatin kidneys as compared with control kidneys, and SAC treatment restored their expressions (Figures 4(A,C)). There was an increase in *G6pc* mRNA expression in mouse kidneys after cisplatin injection, and SAC further increased its expression (Figure 4(B)). Western blot analysis showed decreased renal FBP1 protein levels in Cis group, which were restored by SAC treatment. In contrast, G6PC and PCK1 protein levels remained unchanged in all groups (Figures 4(D–G)). To further assess alterations in glucose metabolism status, we measured glucose and lactose levels both systemically (serum analysis) and locally (renal tissue analysis) in mice. Systemic analysis showed reduced serum glucose and elevated serum lactate in Cis group, indicating that cisplatin-injected mice developed a stress state with suppressed gluconeogenesis. SAC treatment alleviated systemic hypoglycemia and reduced systemic lactate accumulation (Figures 4(H,I)). Further analysis showed that glucose levels in mouse kidney remained stable in all groups, while renal lactate content increased significantly after cisplatin injection and decreased after SAC treatment (Figures 4(J,K)). The coordinated improvement of local and systemic metabolic disturbances underscores the capacity of SAC to reverse stress-induced gluconeogenic suppression, thereby restoring glucose homeostasis.

### *SAC protects HK2 cells from cisplatin-induced injury and rescues gluconeogenic pathway disruption* in vitro

**Figure 5.**
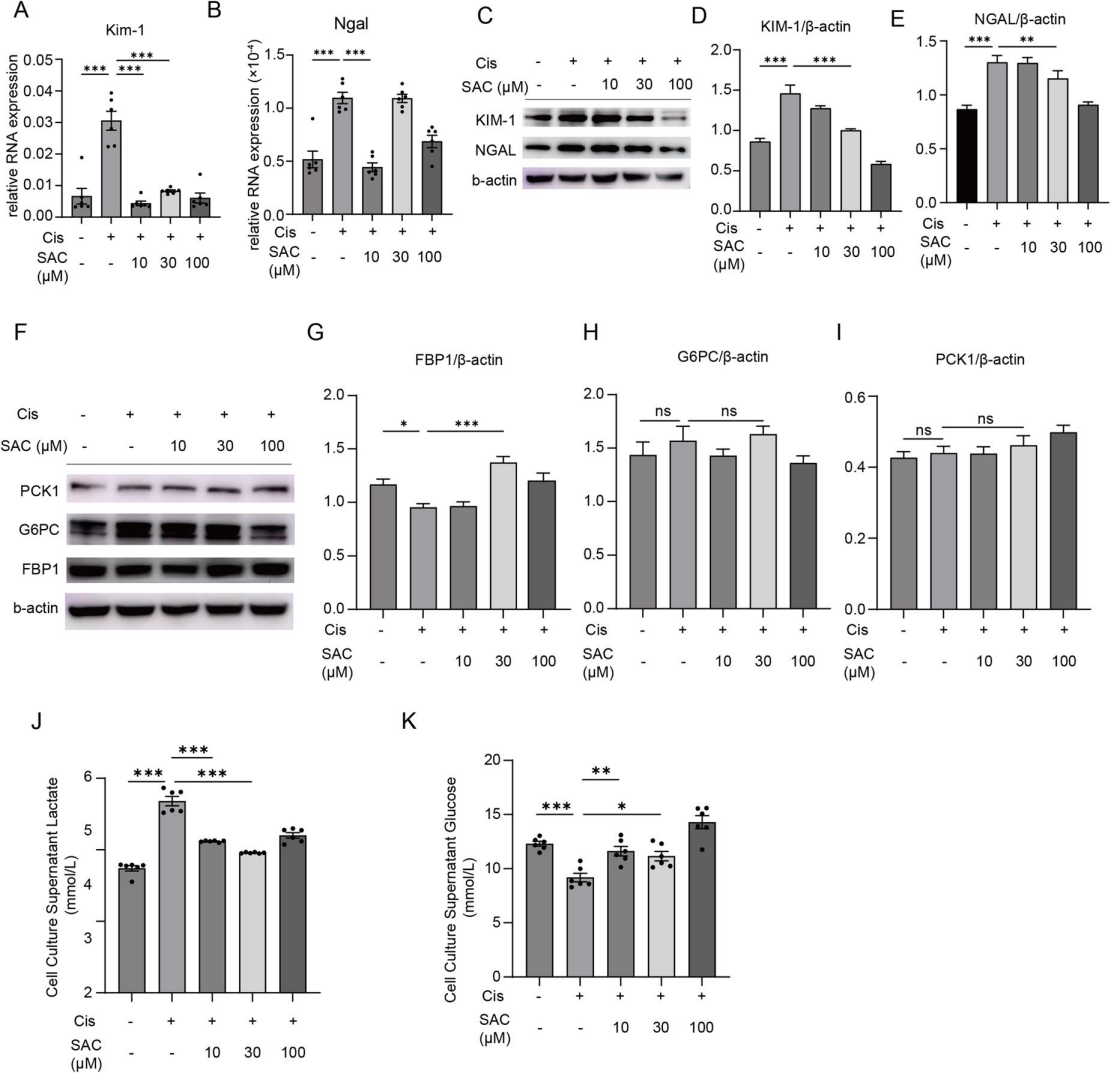
SAC protects HK2 cells from cisplatin-induced injury and rescues gluconeogenic pathway disruption *in vitro*. (A,B) qPCR analysis of *Kim-1*, *Ngal*. (C) Representative Western blot images showing protein expression levels of KIM-1 and NGAL. (D,E) Quantitative analysis of the Western blot results. The bar graphs represent the relative protein levels of KIM-1 and NGAL, calculated as the ratio of the integrated density (IntDen) of each target band to that of the corresponding β-actin band. Data are presented as mean ± SEM. (F) Representative Western blot images showing protein expression levels of FBP1, G6PC, and PCK1. (G–I) Quantitative analysis of the Western blot results. The bar graphs represent the relative protein levels of FBP1, G6PC, and PCK1, calculated as the ratio of the integrated density (IntDen) of each target band to that of the corresponding β-actin band. Data are presented as mean ± SEM. (J,K) Lactate and glucose assessment of cell culture supernatant. NS represents not significant. *p < 0.05. **p < 0.01. ***p < 0.001.

To validate the nephroprotective effects of SAC in a human-relevant *in vitro*, we employed a cisplatin-induced injury model in HK2 cells. Treatment with SAC (10, 30, and 100 μM) dose-dependently attenuated the cisplatin-induced upregulation of the injury markers *Kim-1* and *Ngal* at both mRNA (Figures 5(A,B)) and protein levels (Figures 5(C–E)), with the most pronounced protection observed at 30 μM. We further investigated the impact of SAC on the renal gluconeogenic pathway in this cellular model. Cisplatin exposure significantly suppressed the protein expression of the key gluconeogenic enzyme FBP1, but not G6PC or PCK1 (Figures 5(F–I)). SAC treatment reversed the decrease of FBP1, with 30 μM showing the most robust effect, while 100 μM lost this efficacy (Figure 5(G)). The expression of G6PC and PCK1 proteins remained unchanged across all groups. Furthermore, metabolic analysis of the culture supernatant revealed that cisplatin injury led to a significant accumulation of lactate and decrease of glucose, indicating of a metabolic shift. SAC treatment, particularly at 30 μM, effectively ameliorated these metabolic disturbances, reducing lactate accumulation and restoring glucose levels (Figures 5(J,K)). Collectively, these findings demonstrate that SAC alleviates cisplatin-induced tubular epithelial cell injury, and this protection is associated with the restoration of gluconeogenic flux, potentially through the specific modulation of FBP1. These results align with and strengthen the *in vivo* observations, confirming FBP1-mediated gluconeogenesis as a conserved mechanism underlying SAC’s protection against AKI.

### *FBP1 inhibition reverses the renoprotective effects of SAC* in vivo *and* in vitro

**Figure 6.**
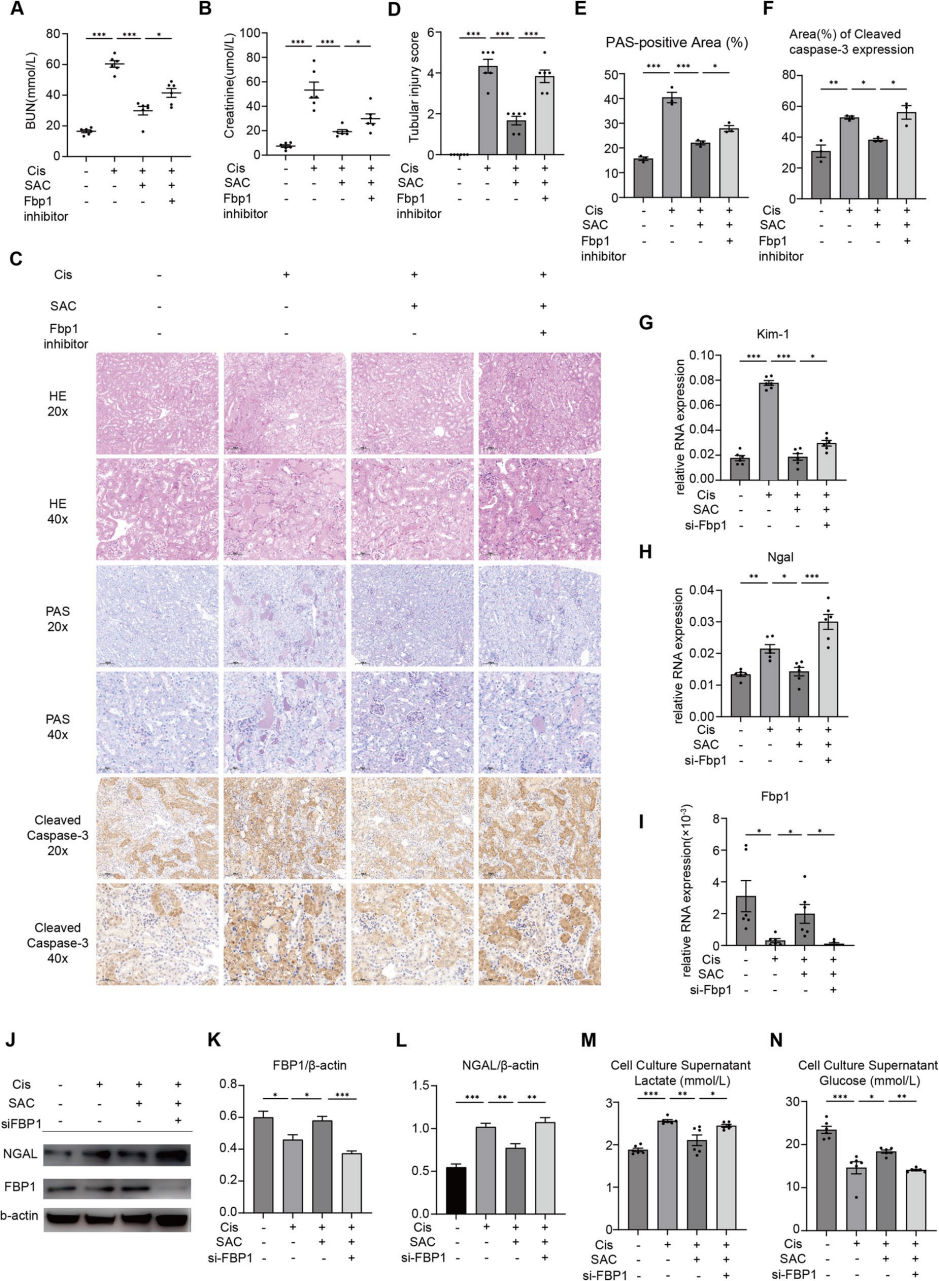
FBP1 inhibition reverses the renoprotective effects of SAC in cisplatin-induced AKI. (A,B) Scr levels and BUN levels. (C) Representative photomicrographs of H&E staining, PAS staining and Cleaved-caspased-3 staining in renal tissues (original magnification ×20, ×40; Scale bar: 100 μm, 50 μm). (D) Quantitative assessment of tubular injury. (E) Quantitative assessment of PAS-positive area. (F) Quantitative assessment of cleaved-caspased-3 Area. (G–I) qPCR analysis of *Kim-1*, *Ngal*, *Fbp1*. (J) Representative Western blot images showing protein expression levels of FBP1 and NGAL. (K,L) Quantitative analysis of the Western blot results. The bar graphs represent the relative protein levels of FBP1 and NGAL, calculated as the ratio of the integrated density (IntDen) of each target band to that of the corresponding β-actin band. Data are presented as mean ± SEM. NS represents not significant. *p < 0.05. **p < 0.01. ***p < 0.001.

Next, we investigated whether FBP1 mediates the protective effect of SAC on AKI *in vivo* by using benzoxazole benzenesulfonamides, a selective FBP1 inhibitor. Figures 6(A,B) showed that SAC inhibited the elevation of Scr and BUN in cisplatin injected mice, which were reversed by FBP1 inhibitor (Figures 6(A,B)). Similarly, FBP1 inhibitor exacerbated tubular injuries in SAC-treated AKI mice as shown by HE staining (Figures 6(C,D)). PAS staining showed that SAC significantly attenuated cisplatin-induced glycogen deposition in renal interstitium, which was nullified by FBP1 inhibition (Figures 6(C,E)). Immunohistochemical analysis further revealed that FBP1 inhibition counteracted SAC’s suppression on cleaved caspase-3 expression (Figures 6(C,F)). To further confirm the effect of FBP1 *in vitro*, we performed an independent set of rescue experiments using FBP1-specific siRNA in HK2 cells. Transfection with FBP1 siRNA effectively knocked down its expression at both mRNA and protein levels (Figures 6(I–K)). Notably, this genetic inhibition of FBP1 reversed the protective effects of SAC against cisplatin-induced injury, proved by the increased expression of injury markers NGAL at both mRNA and protein levels (Figures 6(G,H,J,L)). Moreover, the effects of SAC on the accumulation of lactate and the depletion of glucose in the cell culture supernatant were also reversed upon FBP1 knockdown (Figures 6(M,N)). Thus, our data establish that SAC exerts its renoprotective effects through FBP1.

### SAC-mediated nephroprotection is positively correlated with FOXO1/PGC1α/FBP1 expression

**Figure 7.**
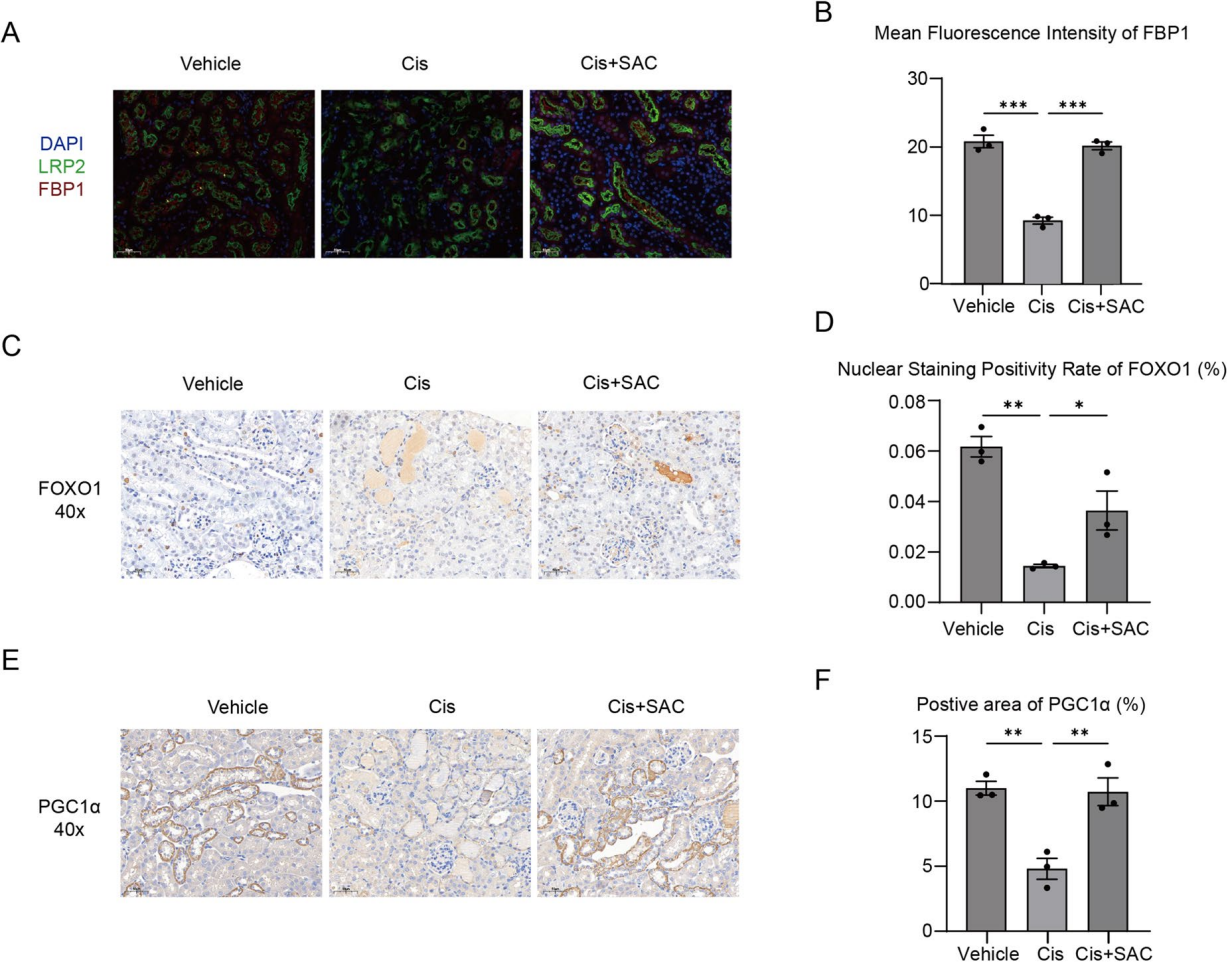
SAC promotes PGC1α/FBP1 axis in AKI mouse kidneys. (A) Representative images of multiplex immunofluorescence staining in renal tissues showing DAPI (blue, nuclei), LRP2 (green, proximal tubules), and FBP1 (red) (original magnification ×40; Scale bar: 50 μm). (B) Quantitative assessment of FBP1-positive area. (C) Representative photomicrographs of FOXO1 staining in renal tissues (original magnification ×40; Scale bar: 50 μm). (D) Quantitative assessment of FOXO1-positive cells. (E) Representative photomicrographs of PGC1α staining in renal tissues (original magnification ×40; Scale bar: 50 μm). (F) Quantitative assessment of PGC1α-positive Area. NS represents not significant. *p < 0.05. **p < 0.01. ***p < 0.001.

Multicolor immunofluorescence staining (DAPI/LRP2/FBP1) analysis revealed that FBP1 was colocalized with LRP2 (a renal tubular marker), and the expression of FBP1 in the cisplatin-induced AKI model was significantly reduced compared to the control group (decreased by 55.50%, *p* < 0.01). However, SAC treatment restored FBP1 expression (2.18-fold increase *vs.* the AKI model group, *p* < 0.01) (Figures 7(A,B)). Further immunohistochemical staining revealed that cisplatin administration decreased total expression of PGC1α and positivity rate of nuclear FOXO1, two upstream regulators of renal gluconeogenesis, in renal tissues. However, SAC intervention significantly reversed these expression patterns (Figures 7(C–F)). These findings establish a positive link between SAC’s renal protection with FOXO1/PGC1α/FBP1 signaling.

### SAC interacts with FBP1

**Figure 8.**
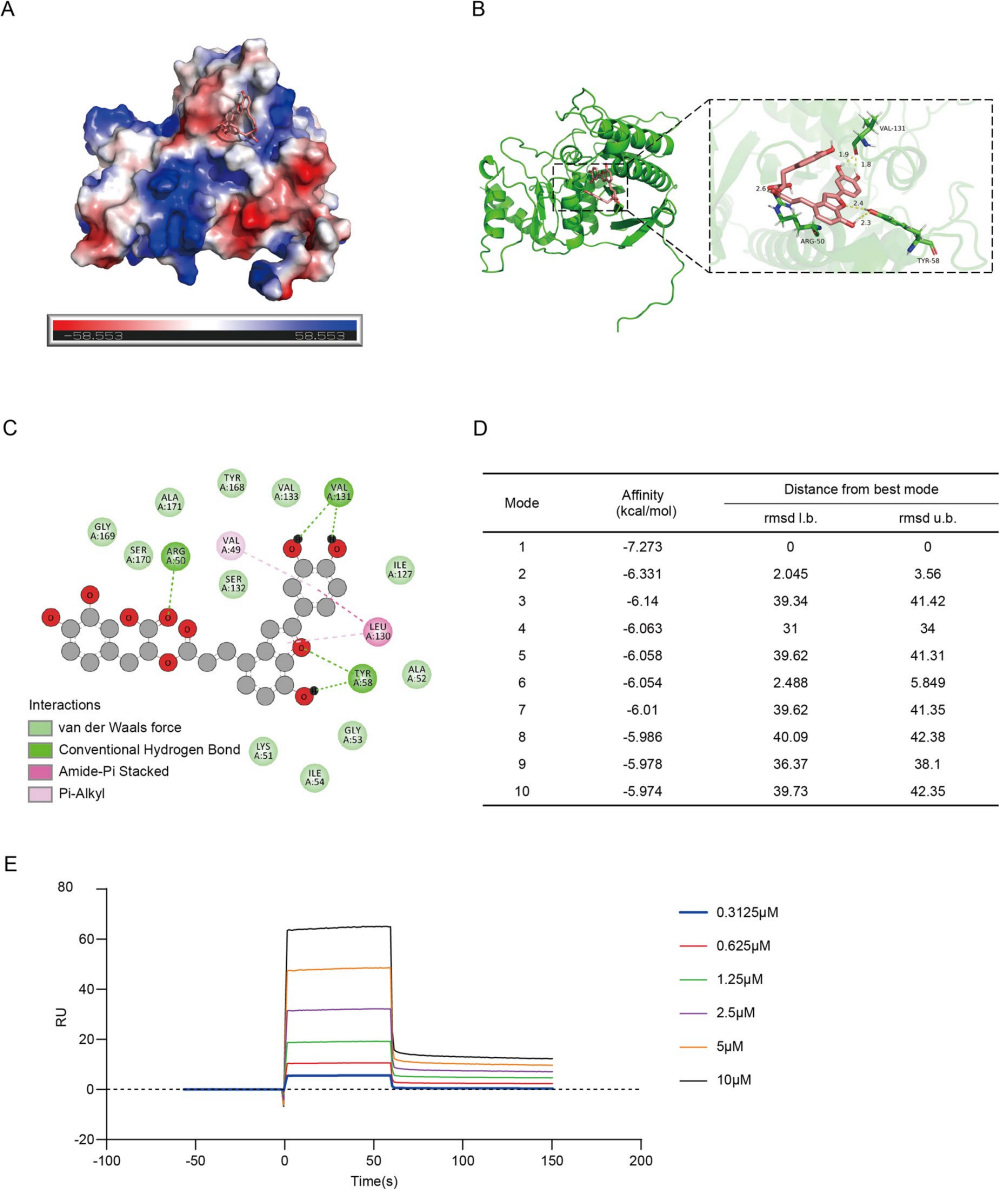
SAC interacts with FBP1 (Uniprot: P09467). Three-dimensional structure of SAC-FBP1 docking is exhibited. (A) 2D structural map of SAC-FBP1 binding. (B) The predicted best mode of SAC and FBP1. (C) The intermolecular forces between SAC and FBP1 binding. (D) The ranking table of molecular docking model binding energies used for different binding sites. (E) SPR binding curve of the interaction between FBP1 protein and SAC.

We next determined whether SAC directly interact with FBP1. Molecular docking analysis revealed a strong binding affinity between SAC and FBP1, with a binding energy of −7.3 kcal/mol (Figure 8(D)). Visualization using PyMOL 3.0.5 and Discovery Studio 2019 demonstrated that SAC binds to the FBP1 active pocket formed by Val-131, Tyr-58, and Arg-50 through hydrogen bonds, π-alkyl interactions (Leu-130), π-stacking (Val-49), and van der Waals forces (Figures 8(A–C)). These interactions stabilize the binding pocket, indicating a potential allosteric mechanism by which SAC enhances FBP1 stability. SPR experiments further validated the direct interaction between SAC and FBP1, yielding an equilibrium dissociation constant (*K_D_*) of 5.17 × 10^−6^ M, an association rate constant (*K_a_*) of 6.98 × 10^4^ Ms^−1^, and a dissociation rate constant (*K_d_*) of 3.61 × 10^−1^ s^−1^. The dose-dependent increase in response units (RU) during the association phase and the slow RU decay during dissociation (Figure 8(E)) aligned with a 1:1 binding model (Langmuir kinetics). These data confirm that SAC directly binds FBP1 with micromolar affinity, which is consistent with the molecular docking predictions.

## Discussion

In our study, we proved that SAC is renal protective in AKI in two different animal models. Through untargeted metabolomics and further validations, the gluconeogenesis pathway was identified as a downstream mechanism underlying the protection of SAC in AKI. Notably, a FBP1 inhibitor completely abolished the therapeutic effects of SAC in cisplatin-induced AKI, suggesting that FBP1 mediates the protective effect of SAC. Molecular docking and SPR assays confirmed direct binding between SAC and FBP1. These findings suggest that FBP1-dependent gluconeogenesis serves as a pivotal regulatory axis in SAC-induced metabolic remodeling during AKI progression.

Our findings identify SAC as one of the natural products that demonstrate therapeutic potential against AKI. Recent studies have highlighted the renoprotective effects of bioactive components from other traditional herbs, such as anthraquinones from *Rheum officinale* and lanostane triterpenoids from *Poria cocos*, which primarily target inflammatory and fibrotic pathways [32,33]. In contrast, the mechanism of limonin and alisol B appears more aligned with cellular signaling modulation, respectively activating ERK2 or inhibiting soluble epoxide hydrolase [34,35]. The specialty of SAC identified herein lies in its direct targeting of a core metabolic enzyme, FBP1, to restore gluconeogenic flux, presenting a distinct mechanism and target among the natural product-based AKI therapies.

This study expands the pharmacological mechanisms of salvianolic acids. Previous researches have predominantly focused on the anti-inflammatory, antioxidant, and anti-fibrotic properties of salvianolic acids [36–39]. While these salutary effects are well-documented, our work shifts the focus to the metabolic dimension. Our study utilized LC-MS/MS-based untargeted metabolomics, from a metabolic perspective, revealing that SAC exerts renoprotective effects in cisplatin-induced AKI by regulating gluconeogenesis and systemic glucose metabolism. Similarly, from a metabolic mechanism standpoint, studies have shown that tanshinone I, another active component of *Salvia miltiorrhiza*, inhibits cancer progression by suppressing glycolysis-related enzymes (HK2/LDHA) and downstream histone lactylation modifications [40]. This mechanism complements our finding that SAC improves AKI by modulating FBP1-dependent gluconeogenesis. Although these two studies target opposing metabolic pathways (inhibition of catabolism *vs.* activation of anabolism), their commonality lies in influencing cellular fate by regulating the activity of core metabolic enzymes related to glucose metabolism. This reflects that SAC may exert therapeutic effects across diverse pathologies *via* precise metabolic reprogramming. Furthermore, the clinical relevance of *S. miltiorrhiza* derivatives is supported by studies on compound Danshen injection, which showed efficacy in improving outcomes in patients with acute cerebral infarction, albeit with a noted low incidence of liver function abnormalities as an adverse effect [41,42]. This clinical context highlights both the translational potential and the need for safety monitoring when developing natural product-based therapies like SAC.

Our study investigated alterations in key gluconeogenic rate-limiting enzymes, including FBP1, G6PC, and PCK1 in cisplatin-induced AKI. The results demonstrated that FBP1 expression was reduced at both mRNA and protein levels, while G6PC decreased only at the mRNA level. Systemic glucose metabolic profiling revealed decreased serum glucose and elevated lactate levels post-cisplatin administration. The suppression of gluconeogenic enzymes and systemic metabolic dysregulation collectively indicated impaired gluconeogenesis, suggesting that glucose metabolism imbalance may represent a critical pathological feature in AKI progression. These findings align with another RNA sequencing data from renal biopsies of early-stage kidney transplant patients, which showed reduced expression of gluconeogenic enzymes (FBP1, PCK1) and increased glycolytic regulators (HK1, PKM, PFKP) during acute ischemic phases post-transplantation [18]. Our study provided a therapeutic option to improve impaired renal glucose metabolism in AKI by which SAC alleviated cisplatin-induced gluconeogenic suppression by upregulating FBP1 expression, enhancing lactate conversion capacity, and reducing renal lactate accumulation.

Importantly, our study further revealed that SAC ameliorates gluconeogenesis during Cis-AKI probably through a dual regulatory mechanism involving the FOXO1/PGC1α/FBP1 signaling axis and direct targeting FBP1. The biological significance of this dual mechanism is profound. Transcriptional upregulation *via* FOXO1/PGC1α ensures a sustained increase in FBP1 synthesis to reprogramme the cellular metabolic state toward gluconeogenesis. Meanwhile, direct binding to FBP1 may enhance its catalytic efficiency or protect it from degradation under stress, providing an immediate post-translational boost to enzymatic activity. This may create a robust mechanism to rapidly restore glucose homeostasis in injured tubules, a critical advantage in the dynamic and fragile context of AKI. The uniqueness of SAC, compared to general transcriptional activators or allosteric enzyme modulators, lies in this integrated capacity to hit the same key molecular target (FBP1) at two distinct regulatory levels, potentially yielding a more potent and specific therapeutic effect.

FBP1 is involved in diverse metabolic disorders and cancers. For example, hepatocyte-specific FBP1 deficiency induces steatosis with hepatic stellate cell activation and senescence [43], while also causing fructose intolerance under high-fructose diet [44]. In pancreatic β-cells, FBP1 modulates insulin secretion, with overexpression impairing glucose metabolism and insulin release [45,46]. Diabetic nephropathy patients exhibit elevated urinary FBP1 levels but reduced renal tubular FBP1 expression, suggesting its biomarker potential [47]. Conversely, ovarian cancer cells with FBP1 knockout display enhanced cisplatin sensitivity *via* EZH2 downregulation and reduced H3K27me levels [48]. Remarkably, a study in *Nature* revealed that in metabolic dysfunction associated steatohepatitis, FBP1 is a p53-induced senescence marker, and its subsequent loss *via* an AKT/NRF2-driven switch is critical for hepatocarcinogenesis [49]. This highlights the critical role of FBP1 as a tumor suppressor in the liver, where its loss promotes proliferation. Furthermore, a recent study implicated reduced FBP1 expression in polystyrene nanoplastic-induced cardiac injury *via* a gut-heart axis, suggesting its role in metabolic communication between organs [50]. Unlike these paradigms where FBP1 inhibition is sought (in diabetes) or its loss is pathogenic (e.g., cancer/cardiac injury), where tubular energetics collapse and lactate accumulates in AKI, enhancing FBP1-mediated gluconeogenesis serves as a rescue mechanism to clear toxic metabolites and regenerate energy, distinguishing it from approaches aimed at suppressing an overactive pathway.

An important observation in our study was the discordant regulation of G6pcand Pck1mRNA *versus* their protein levels following cisplatin or SAC treatment. While *Pck1*mRNA was elevated, its protein abundance remained unchanged. Such discrepancies hint at the potential involvement of post-transcriptional regulatory mechanisms gluconeogenesis. In metabolic diseases, the expression of key enzymes like G6PC is known to be regulated by epitranscriptomic modifications, such as N6-methyladenosine on its mRNA, which can modulate transcript stability and translation efficiency independently of transcription levels [51]. Furthermore, microRNA targeting upstream transcriptional regulators such as the KDM2A/FOXO1 axis can also dissociate mRNA expression from functional protein output for downstream targets, including PCK1 and G6PC [52]. Although the specific post-transcriptional layer operating in our AKI model warrants future investigation, our data identify FBP1 as the pivotal and consistently regulated factor through which SAC restores gluconeogenesis and exerts renoprotection.

Thus, intervention strategies based on FBP1 has been reported in different diseases. Hepatic FBP1 degradation *via* Protein Kinase B (AKT)/Nuclear Factor Erythroid 2 related factor 2 (NRF2) accelerates precancerous hepatocyte proliferation [49]. On the contrary, in diabetic model, FBP1 inhibitor can inhibit pathological gluconeogenesis, and based on this target, many anti-diabetic drugs have been developed [53–55]. Unlike these studies on FBP1 inhibition, we identified a strategy to promote FBP1 signaling by SAC treatment in AKI.

Our molecular docking and SPR data confirm a direct physical interaction between SAC and FBP1. The key residues of human FBP1 active site have been reported locating on Asn212, Tyr215, Tyr244, etc. [56], that directly coordinate the substrate/product. By comparing this canonical active site with our docking results, we can infer that SAC binds to a distinct yet adjacent pocket involving residues Val-131, Tyr-58, and Arg-50. The proximity to the catalytic core means the interaction with this region allows SAC to potentially influence the active site’s geometry or dynamics without competing for substrate binding. Notably, this binding site of SAC is also distinct from the canonical allosteric inhibitory site occupied by AMP (which involves residues such as Tyr113 and Arg140) [57], indicating that SAC does not act as a substrate analog or a competitor at the AMP allosteric site. Instead, it likely functions as a positive modulator that may stabilize the function form of FBP1. The SPR-determined equilibrium dissociation constant (KD) of 5.17 µM indicates a direct and specific interaction with micromolar affinity. In drug discovery, such affinity is considered favorable for natural product leads. Crucially, this KD value also aligns pharmacologically with the effective concentrations of SAC (10–30 µM) observed in our *in vitro* experiments. Furthermore, the effective *in vivo* dose (10 mg/kg) in our mouse models is anticipated to achieve tissue concentrations within this micromolar range, supporting the physiological relevance of the observed interaction.

### Limitations

Despite the novel findings, this study has several limitations. First, all experiments *in vivo* were conducted using male mice, which precludes the evaluation of potential sex-dependent differences in SAC’s efficacy or the FBP1-mediated mechanism. Second, the pharmacokinetic profile of SAC, including its absorption, distribution, metabolism, and excretion in the context of AKI, remains uncharacterized and is crucial for understanding its therapeutic window and translational potential. Third, while SAC demonstrated efficacy in acute injury models, its long-term benefits and capacity to prevent the transition from AKI to CKD were not explored. Fourth, detailed mechanistic validation in IRI models is lacking to confirm the generalizability of the FBP1-dependent mechanism across AKI etiologies. Finally, our current data demonstrate that SAC binds to and upregulates FBP1, but direct evidence that SAC modulates FBP1 enzymatic activity awaits further biochemical validation. Addressing these points will be a focus of subsequent research.

## Conclusion

SAC attenuates AKI by promoting FBP1-mediated renal gluconeogenesis.

## Supplementary Material

Supplemental Material

## Data Availability

The authors confirm that the data supporting the findings of this study are available within the article and its Supplementary Materials.
