# Supplementary material for: Salvianolic acid C alleviates acute kidney injury by restoring fructose-1,6-bisphosphatase 1-mediated gluconeogenesis
Source: Ren Fail. 2026 Mar 8;48(1):2629902. doi: 10.1080/0886022X.2026.2629902 (PMC12973791; doi:10.1080/0886022X.2026.2629902)
Supplement: Supplemental Material [file IRNF_A_2629902_SM9582.docx]

**Supplementary materials**

**This file includes:**

**Supplementary figures: Supplementary figure A1- A10.**

**Supplementary tables: Supplementary table B1- B.**

**Supplementary figures**

Supplementary figure A1. Original Image for Fig1.D


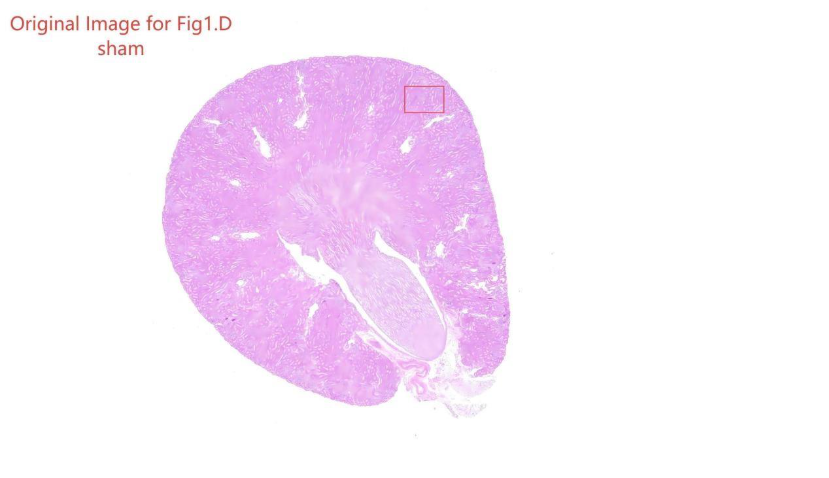


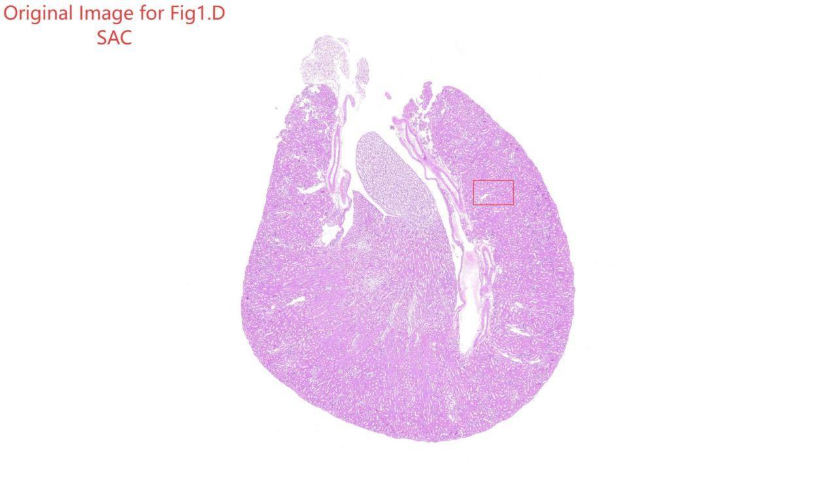


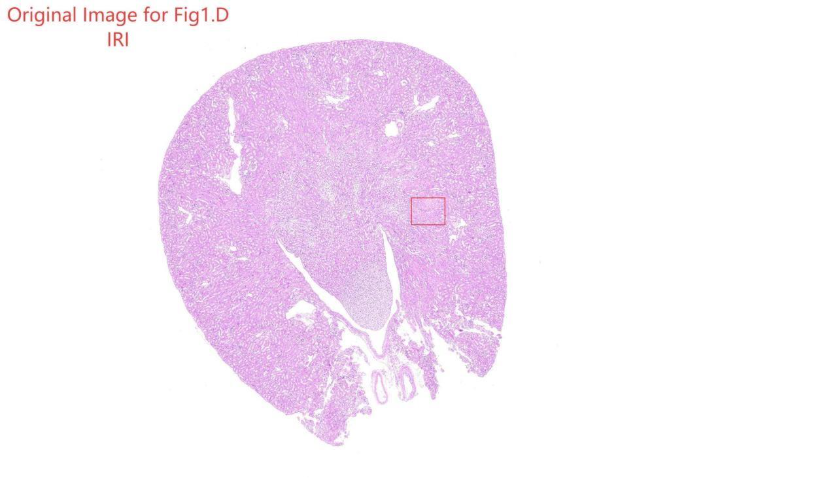

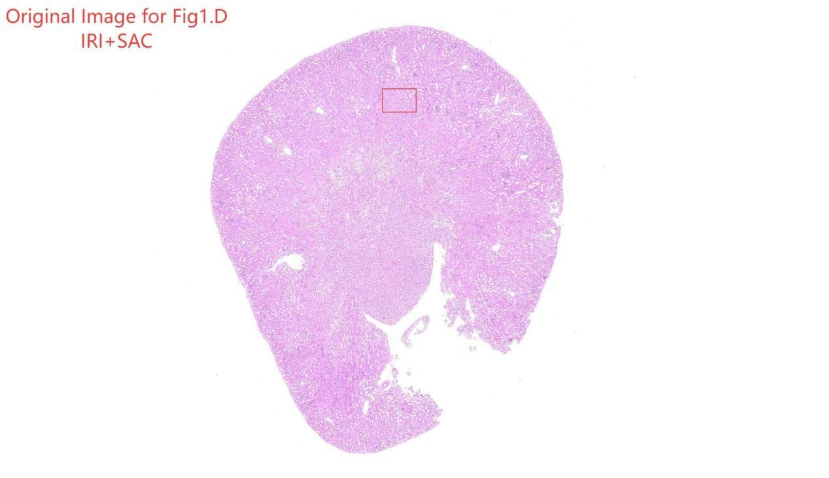


Supplementary figure A2.Original Image for Fig1.I


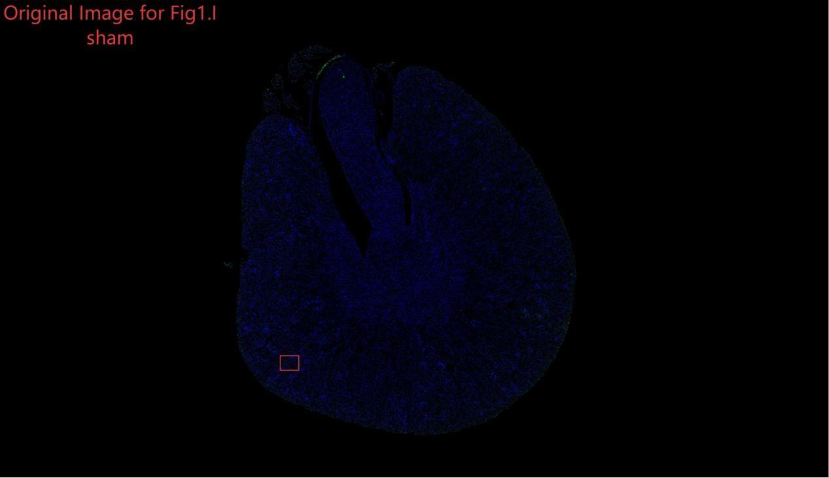

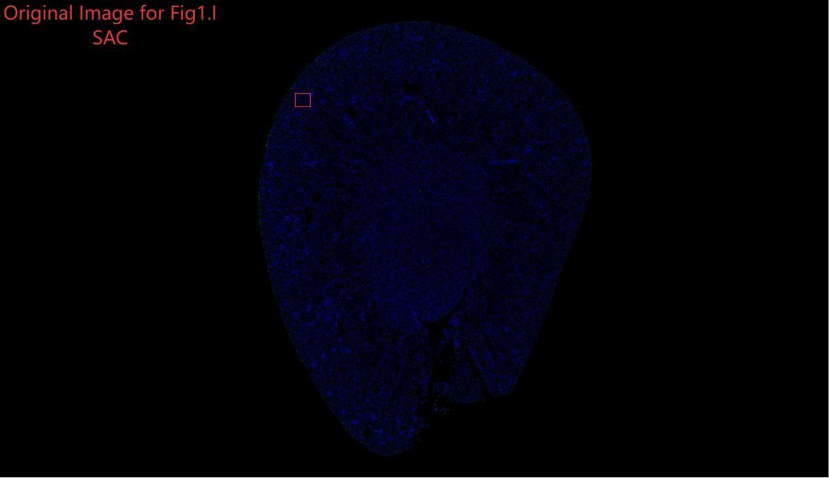

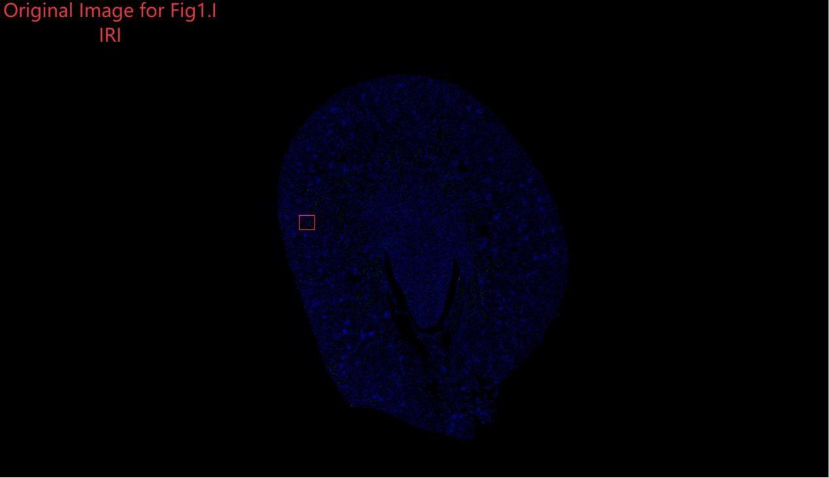

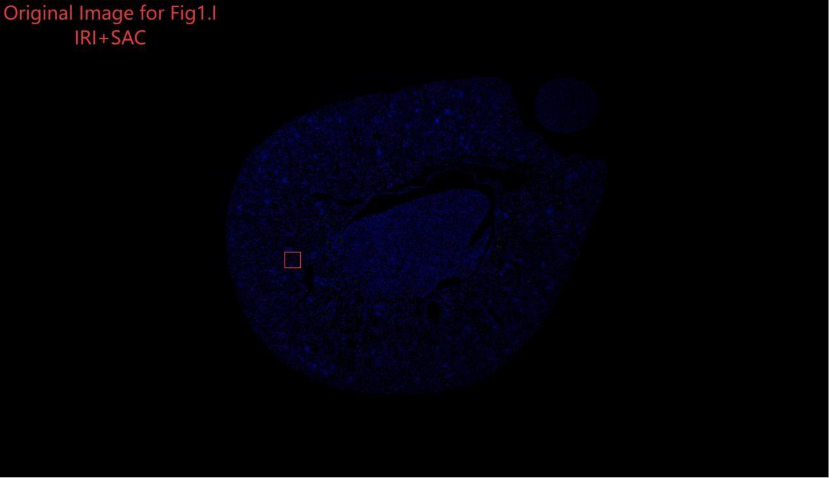


Supplementary figure A3. Original Image for Fig2.D


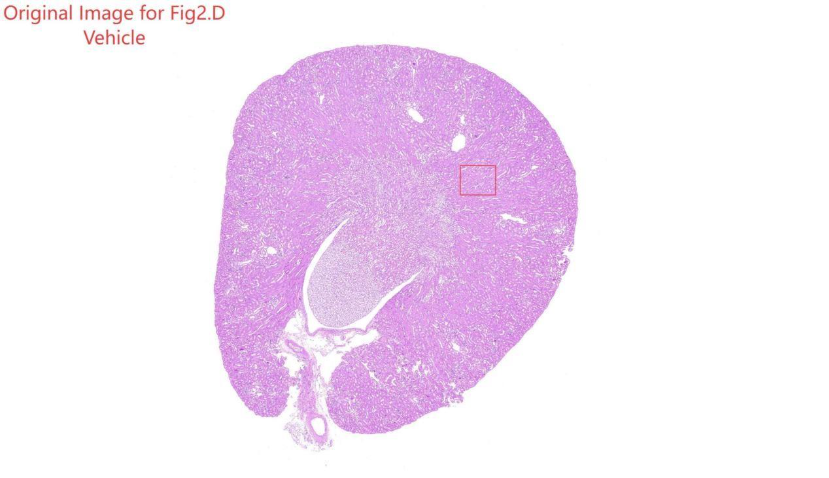


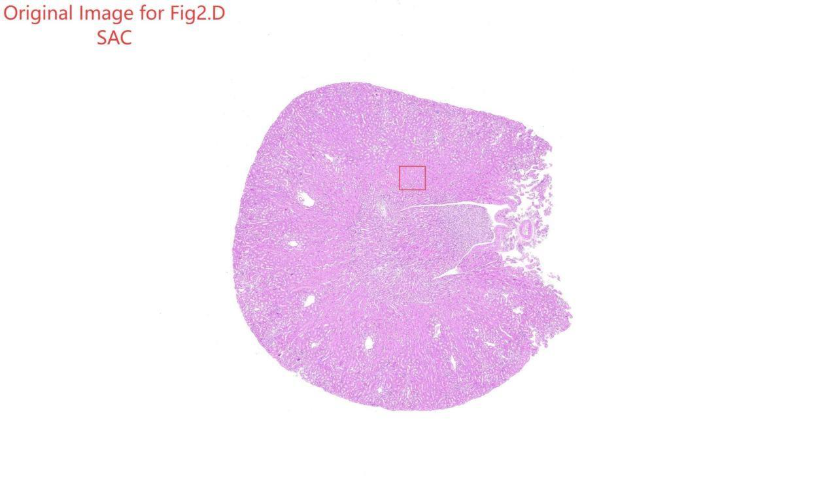


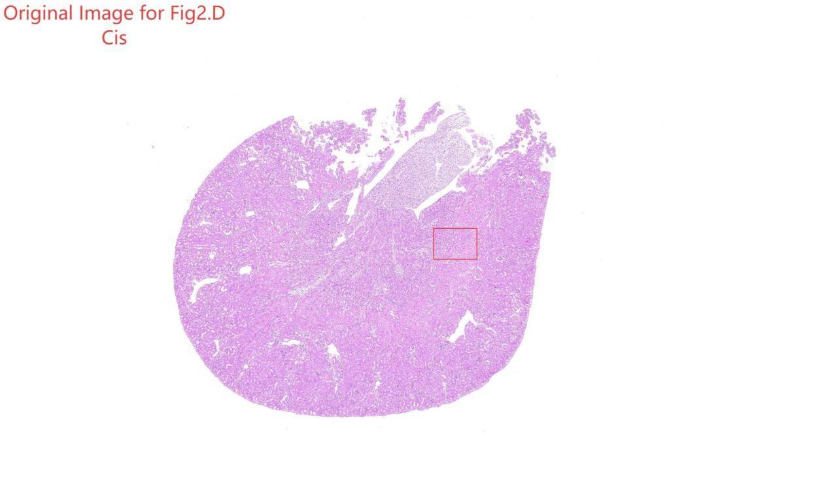


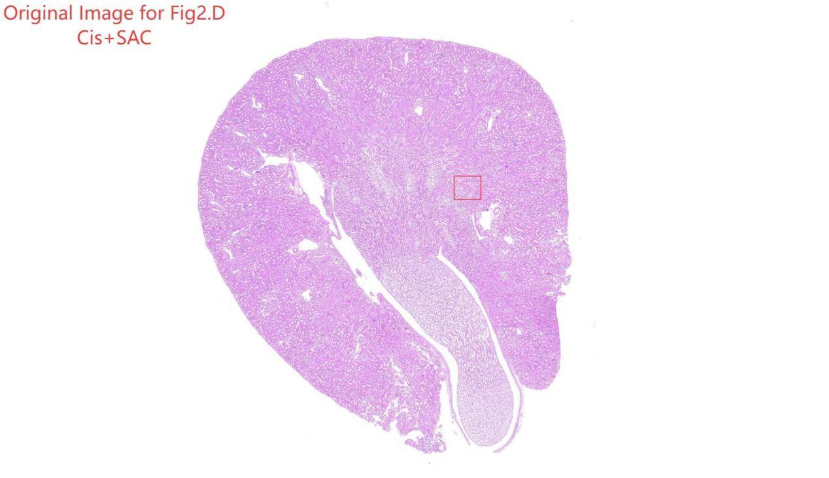


Supplementary figure A4. Original Image for Fig2.I


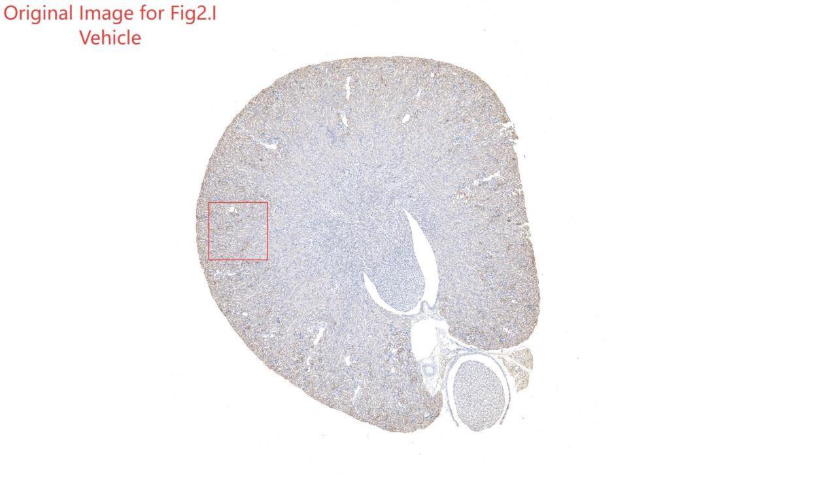


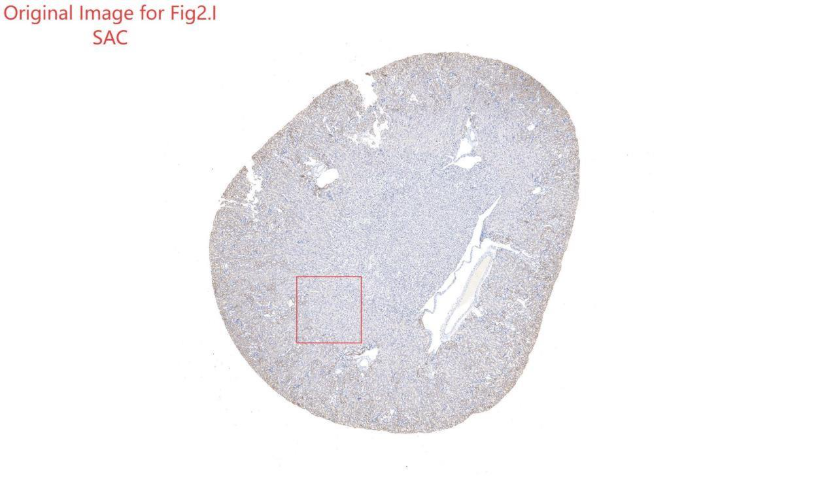


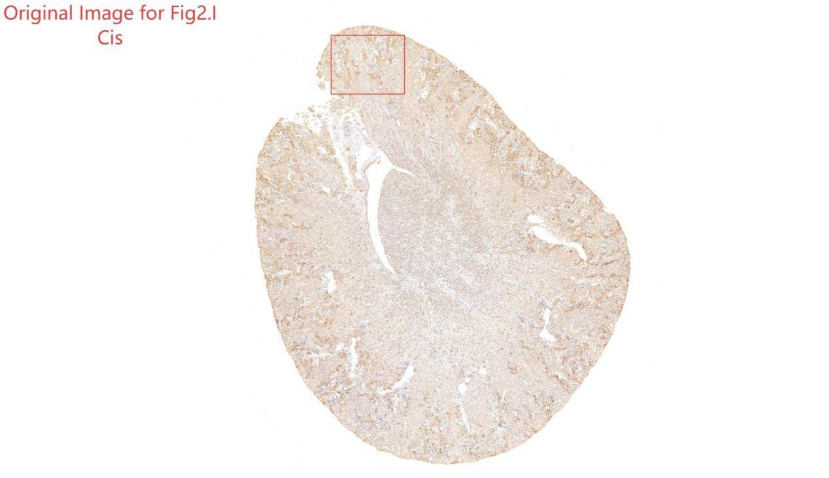


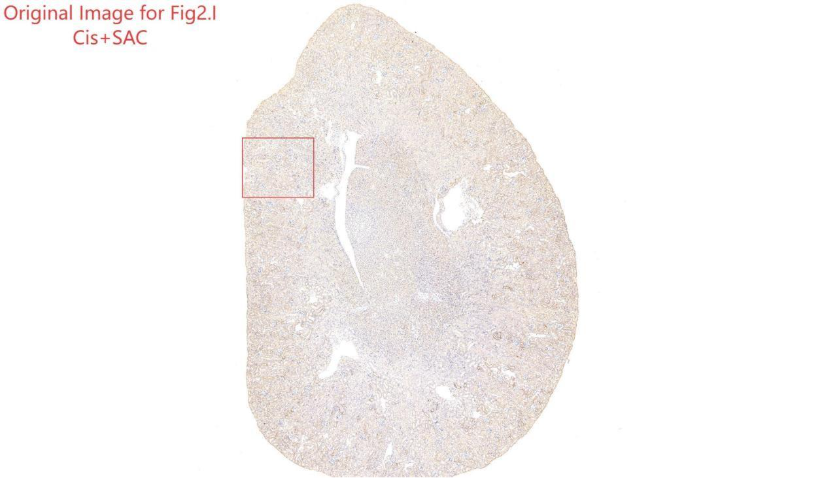


Supplementary figure A5. Original Image for Fig5.C (HE staining).


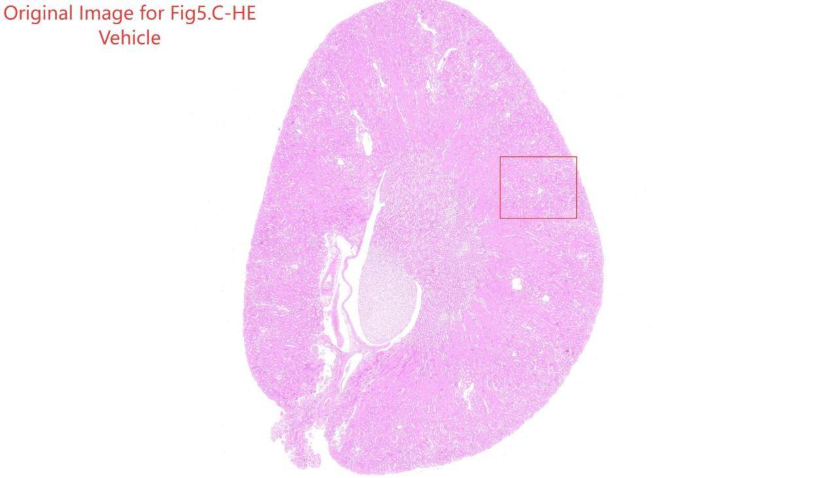


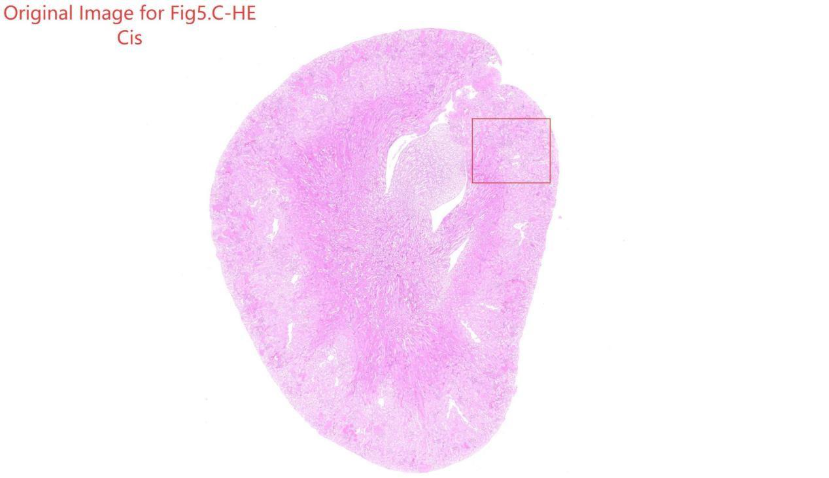


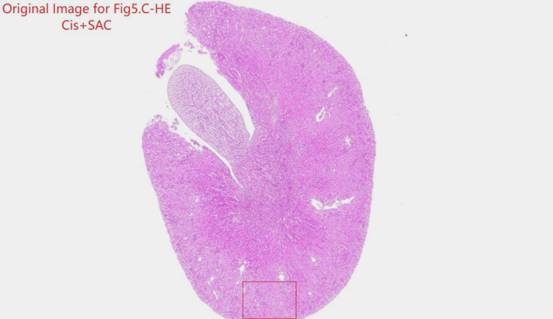


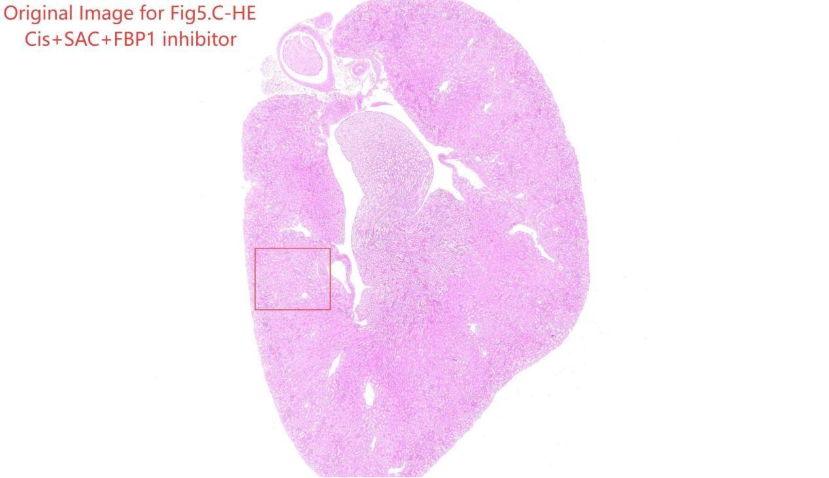


Supplementary figure A6. Original Image for Fig5.C (PAS staining).


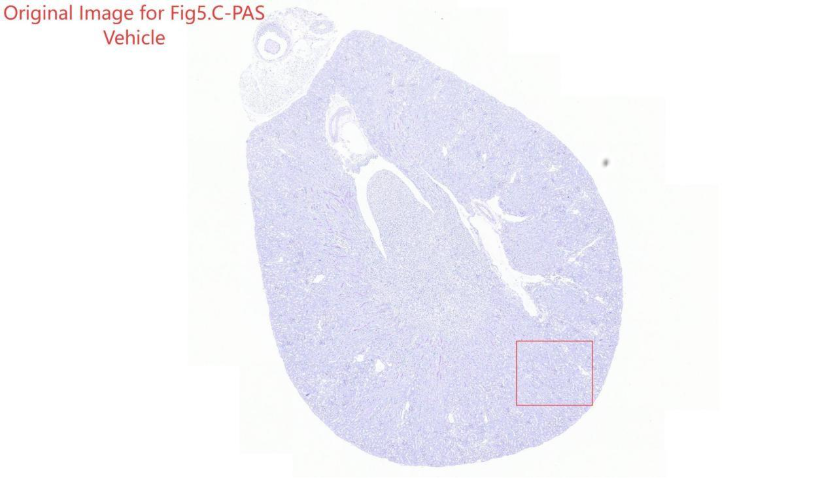


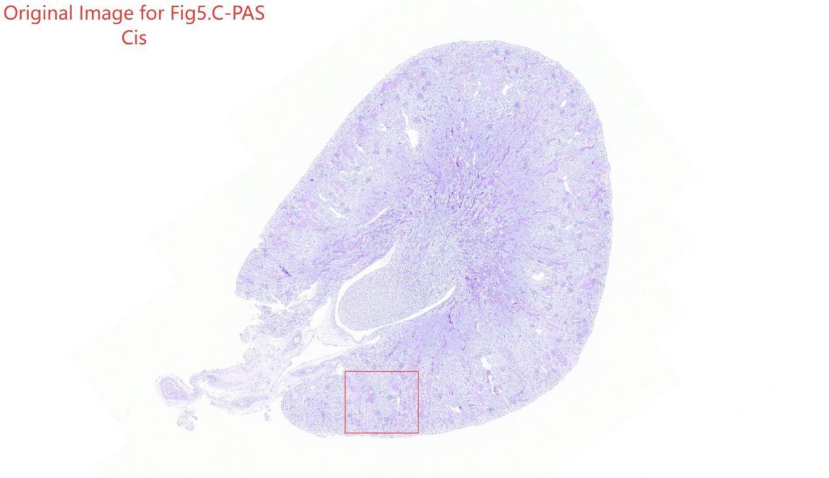


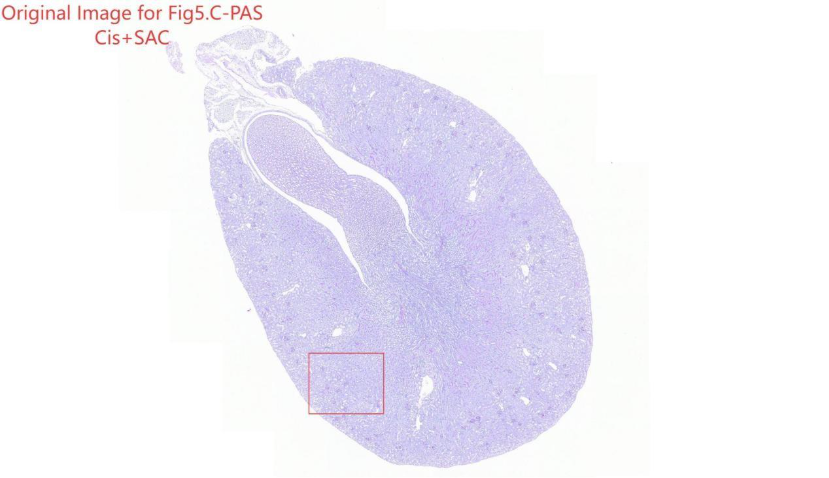

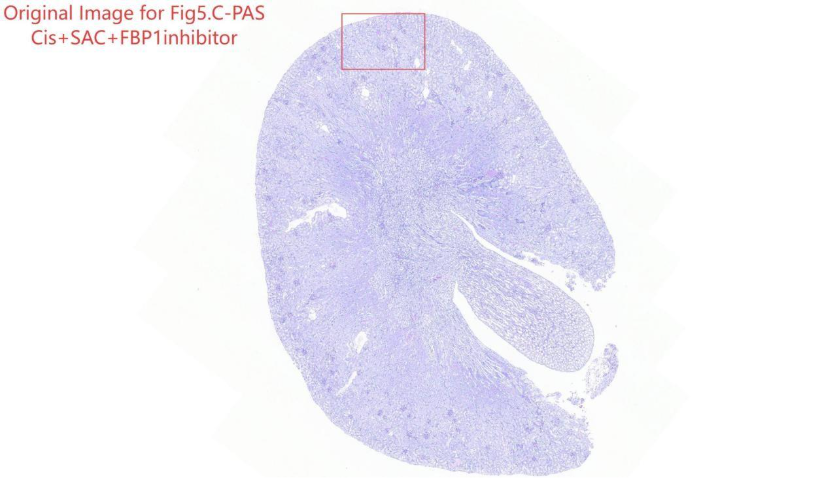


Supplementary figure A7. Original Image for Fig5.C (Cleaved-caspased-3 staining)


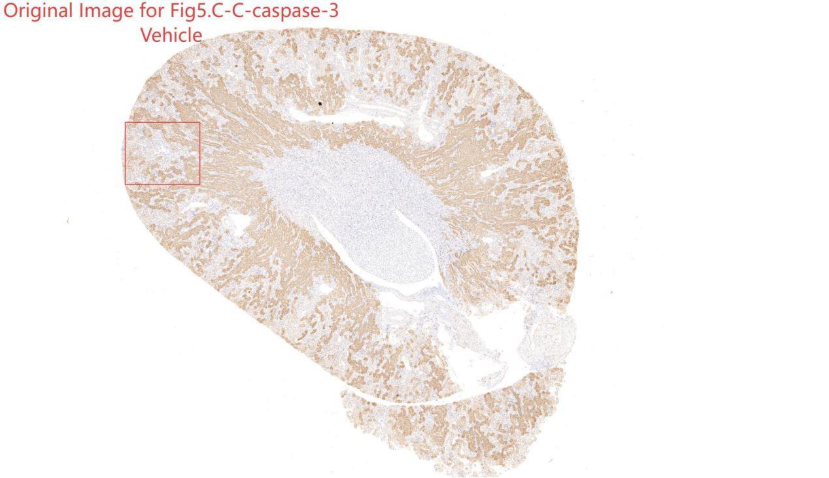


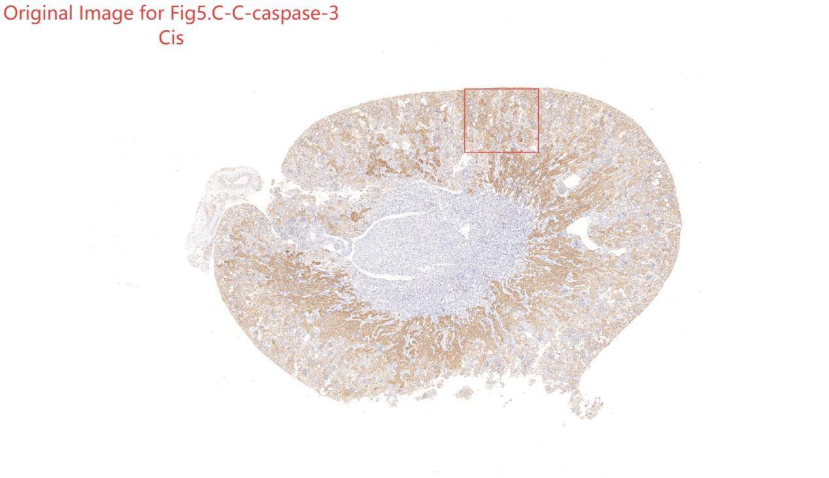

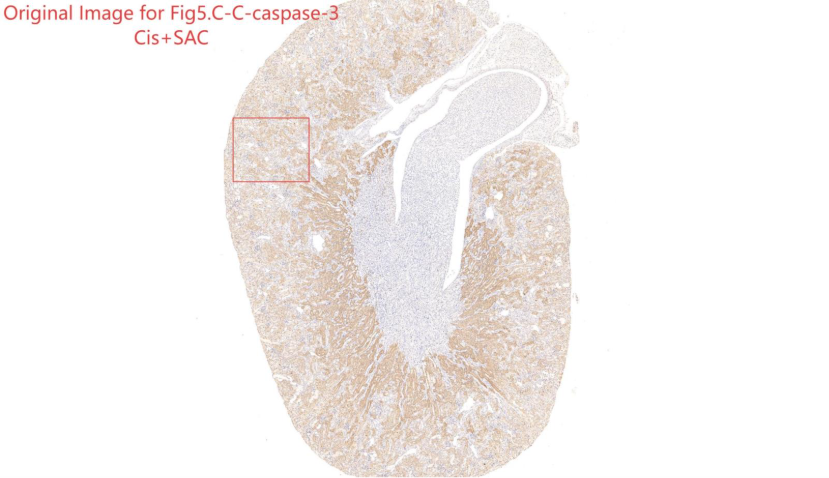


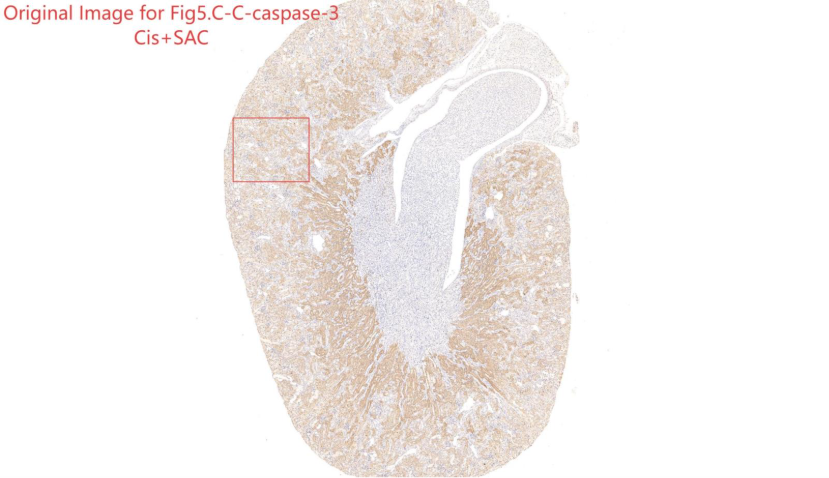


Supplementary figure A8. Original Image for Fig6.A


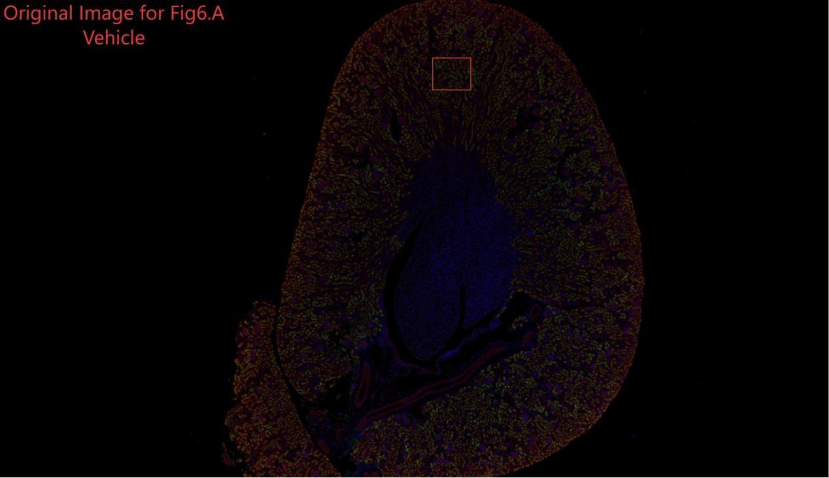

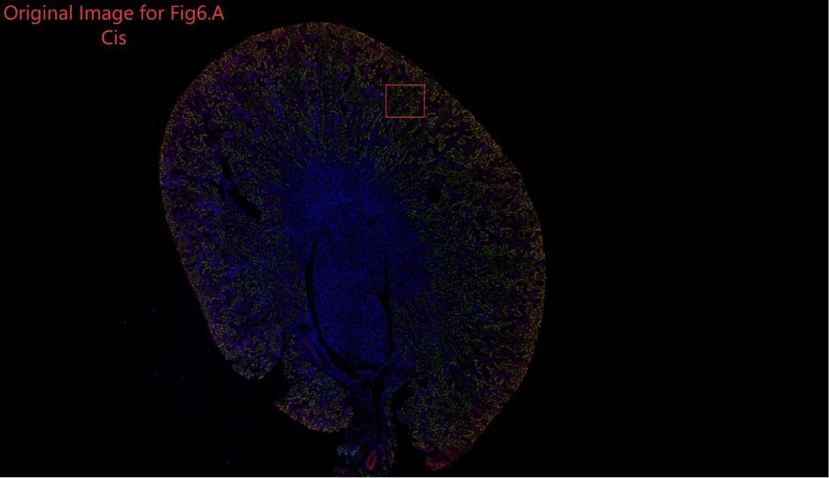

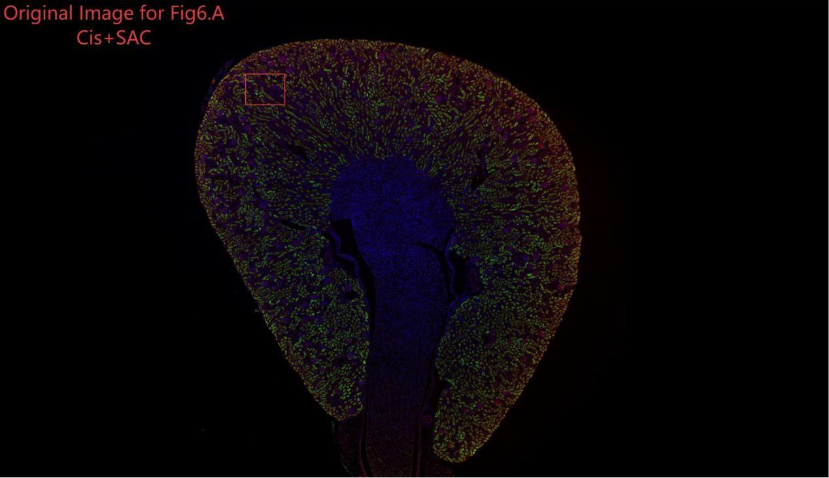


Supplementary figure A9. Original Image for Fig6.C


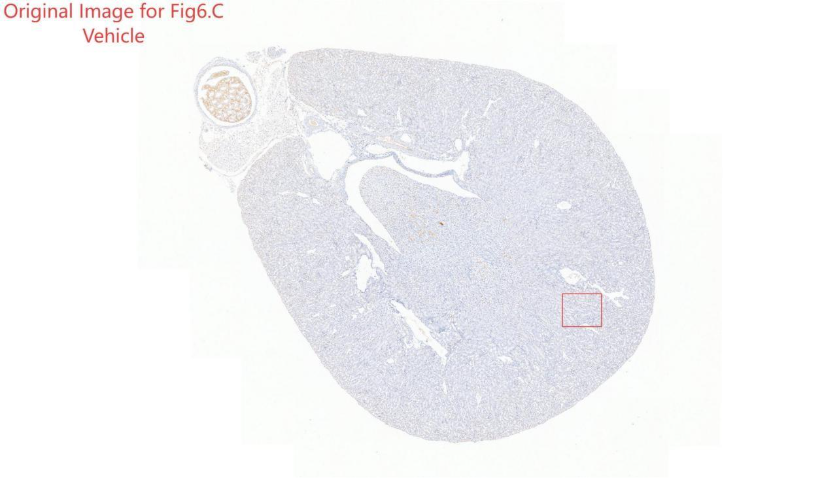


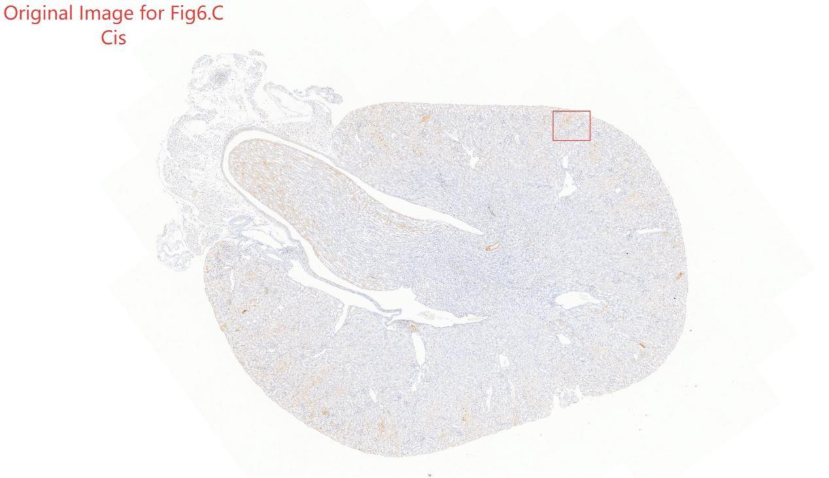


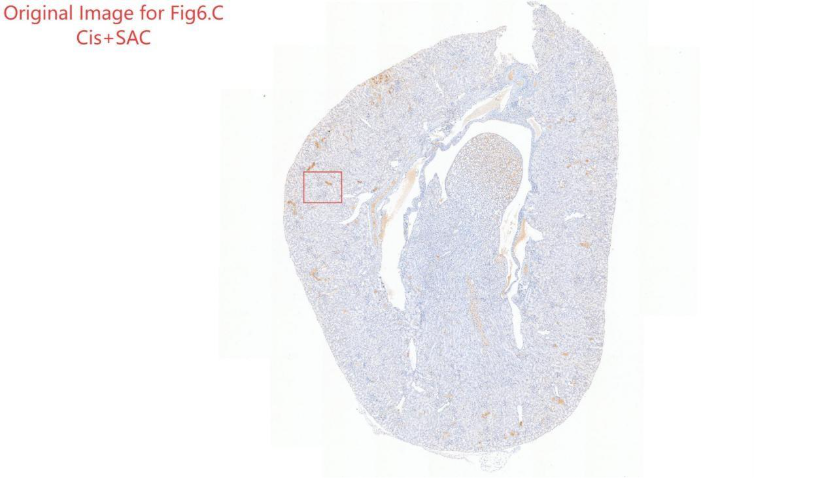


Supplementary figure A10. Original Image for Fig6.E


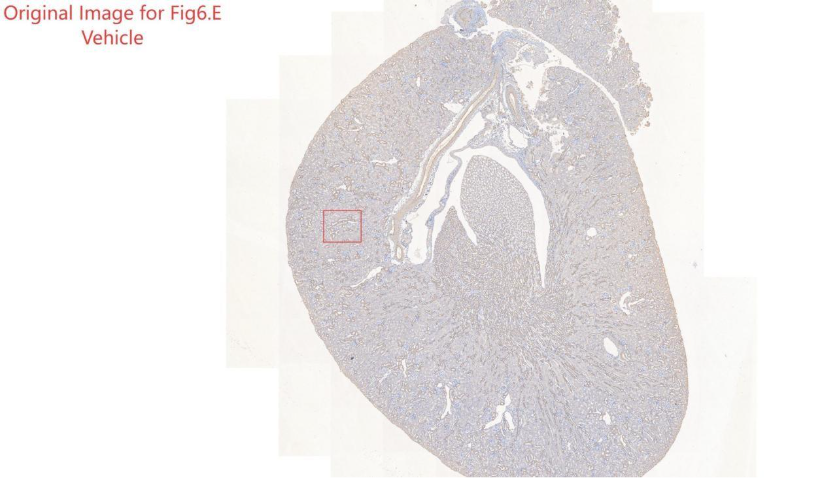

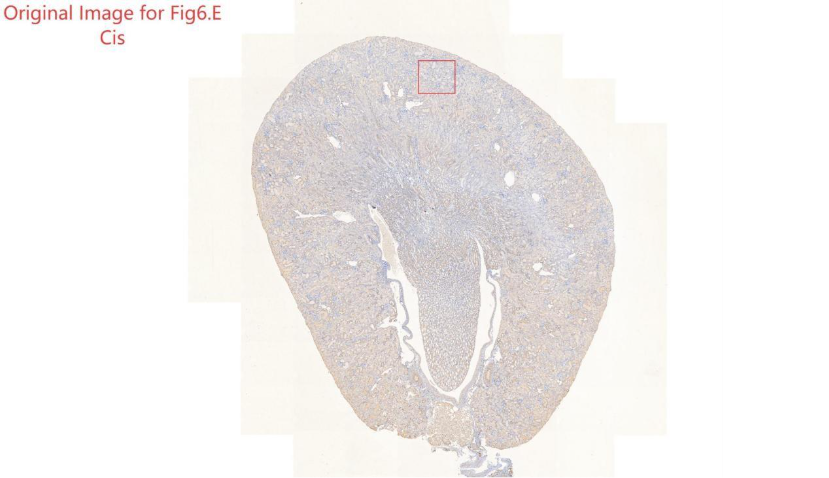

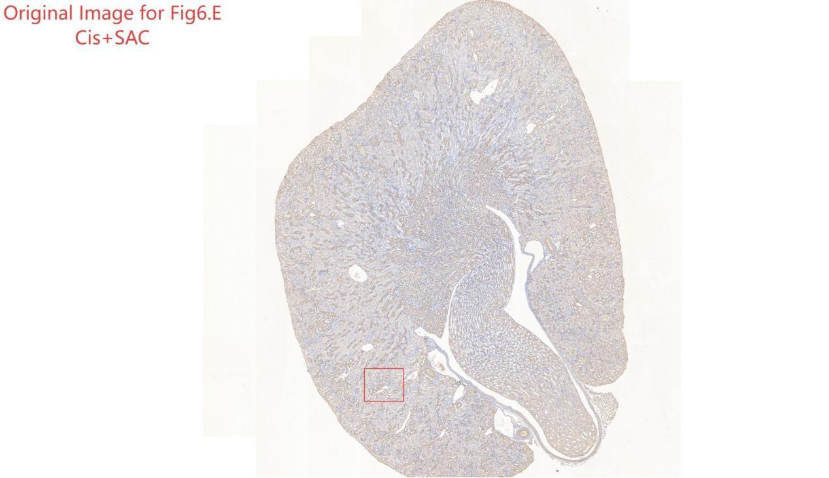


B. Supplementary tables: Supplementary table B1- B13

Supplementary tableB1. Information of Key Reagents and Kits

| **Reagent/Kit Name** | **Catalog Number** | **Manufacturer** | **Location** |
| --- | --- | --- | --- |
| Salvianolic acid C | T3149 | TargetMol | Shanghai, China |
| Scr Assay Kit | C011-2-1 | Nanjing Jiancheng Bioengineering Institute | Nanjing, Jiangsu, China |
| BUN Assay Kit | C013-2-1 | Nanjing Jiancheng Bioengineering Institute | Nanjing, Jiangsu, China |
| Lactate Assay Kit | A019-2-1 | Nanjing Jiancheng Bioengineering Institute | Nanjing, Jiangsu, China |
| Glucose Assay Kit | A154-1-1 | Nanjing Jiancheng Bioengineering Institute | Nanjing, Jiangsu, China |
| RNA Rapid Extraction Kit | RN001 | ESscience Biotech | Shanghai, China |
| SYBR Green premix | RR 420A | Takara Bio | Shiga, Japan |
| TB Green Premix Ex Taq | RR820A | Takara Bio | Shiga, Japan |
| RIPA lysis buffer | P0013B | Beyotime Biotechnology | Shanghai, China |
| BCA protein assay kit | WB6501 | New Cell & Molecular Biotech | Suzhou, Jiangsu, China |
| SurePAGE™, Bis-Tris, 8% | M00657 | GenScript Biotech | Nanjing, Jiangsu, China |
| PVDF membrane | L00733C/L00734C | GenScript Biotech | Nanjing, Jiangsu, China |
| TBST Buffer | C520009 | Sangon Biotech | Shanghai, China |
| ECL reagent | WBKLS0500 | Merck Millipore | Burlington, MA, USA |
| Hematoxylin-Eosin Staining Kit | G1076 | Servicebio | Wuhan, Hubei, China |
| PAS Staining Kit | G1008 | Servicebio | Wuhan, Hubei, China |
| Proteinase K | G1205 | Servicebio | Wuhan, Hubei, China |
| TUNEL Kit (TMR Red) | G1502 | Servicebio | Wuhan, Hubei, China |
| LipofectamineTM 3000 | L3000001 | Thermo Fisher Scientific | Carlsbad, California, USA |
| Zoletil 50 | Zoletil 50 | Hangzhou Shuo Ye | Hangzhou, China |
| Dexmedetomidine hydrochloride | 2011 | Hangzhou Shuo Ye | Hangzhou, China |

Supplementary tableB2. Information of Key Instruments and Software

| **Instrument/Software Name** | **Manufacturer** | **Location** |
| --- | --- | --- |
| NIKON ECLIPSE E100 Upright Microscope | Nikon | Tokyo, Japan |
| Fluorescence Microscope (Nikon Eclipse C1) | Nikon | Tokyo, Japan |
| UHPLC-Q Exactive HF-X Mass Spectrometer | Thermo Fisher Scientific | Waltham, MA, USA |
| ImageJ Software | Media Cybernetics | Rockville, MD, USA |
| Majorbio Cloud Platform | Majorbio | Shanghai, China |

Supplementary tableB3. Original Integrated Density of β-actin and NGAL for Fig.1G

|  | sham | SAC | IRI | IRI+SAC |
| --- | --- | --- | --- | --- |
| Bactin | 584474.00 | 648477.00 | 595429.00 | 646923.00 |
|  | 639265.00 | 582515.00 | 602208.00 | 614765.00 |
|  | 612081.00 | 588197.00 | 587946.00 | 612581.00 |
|  | 611315.00 | 618142.00 | 591218.00 | 624438.00 |
| NGAL | 444208.00 | 338594.00 | 848650.00 | 263118.00 |
|  | 383224.00 | 244298.00 | 762623.00 | 316128.00 |
|  | 347306.00 | 322545.00 | 802395.00 | 403193.00 |
|  | 307704.00 | 291295.00 | 813595.00 | 302502.00 |
| NGAL/Bactin | 0.76 | 0.52 | 1.43 | 0.41 |
|  | 0.60 | 0.42 | 1.27 | 0.51 |
|  | 0.57 | 0.55 | 1.36 | 0.66 |
|  | 0.50 | 0.47 | 1.38 | 0.48 |

Supplementary B4. Original Integrated Density of β-actin and Bax/Bcl-2 for Fig.1H

|  | sham | SAC | IRI | IRI+SAC |
| --- | --- | --- | --- | --- |
| Bactin | 584474.00 | 582515.00 | 595429.00 | 614765.00 |
|  | 639265.00 | 588197.00 | 587946.00 | 612581.00 |
|  | 611315.00 | 618142.00 | 591218.00 | 624438.00 |
| Bax | 440339.00 | 734455.00 | 687116.00 | 461754.00 |
|  | 435829.00 | 617688.00 | 664758.00 | 383337.00 |
|  | 537656.00 | 594440.00 | 759415.00 | 385734.00 |
| Bax/Bactin | 0.75 | 1.26 | 1.15 | 0.75 |
|  | 0.68 | 1.05 | 1.13 | 0.63 |
|  | 0.88 | 0.96 | 1.28 | 0.62 |
| Bcl-2 | 293053.00 | 501864.00 | 447533.00 | 646508.00 |
|  | 229341.00 | 445999.00 | 430919.00 | 592023.00 |
|  | 368990.00 | 440310.00 | 400540.00 | 440304.00 |
| Bcl-2/Bactin | 0.50 | 0.86 | 0.75 | 1.05 |
|  | 0.36 | 0.76 | 0.73 | 0.97 |
|  | 0.60 | 0.71 | 0.68 | 0.71 |
| Bax/Bcl-2 | 1.50 | 1.46 | 1.54 | 0.71 |
|  | 1.90 | 1.38 | 1.54 | 0.65 |
|  | 1.46 | 1.35 | 1.90 | 0.88 |

Supplementary B5. Original Integrated Density of β-actin and NGAL for Fig.2G

|  | Vehicle | SAC | Cis | Cis+SAC |
| --- | --- | --- | --- | --- |
| Bactin | 307393.00 | 328464.00 | 354926.00 | 332623.00 |
|  | 326906.00 | 347351.00 | 370514.00 | 350580.00 |
|  | 293409.00 | 318976.00 | 321804.00 | 263438.00 |
|  | 335807.00 | 334131.00 | 327846.00 | 288978.00 |
| NGAL | 102898.00 | 83099.00 | 804785.00 | 364895.00 |
|  | 95045.00 | 71952.00 | 749374.00 | 345712.00 |
|  | 119370.00 | 106393.00 | 752883.00 | 88315.00 |
|  | 118542.00 | 84934.00 | 737303.00 | 76453.00 |
| NGAL/Bactin | 0.33 | 0.25 | 2.27 | 1.10 |
|  | 0.29 | 0.21 | 2.02 | 0.99 |
|  | 0.41 | 0.33 | 2.34 | 0.34 |
|  | 0.35 | 0.25 | 2.25 | 0.26 |

Supplementary B6. Original Integrated Density of β-actin and KIM-1 for Fig.2H

|  | Vehicle | SAC | Cis | Cis+SAC |
| --- | --- | --- | --- | --- |
| Bactin | 307393.00 | 328464.00 | 354926.00 | 332623.00 |
|  | 326906.00 | 347351.00 | 370514.00 | 350580.00 |
|  | 293409.00 | 318976.00 | 321804.00 | 263438.00 |
|  | 335807.00 | 334131.00 | 327846.00 | 288978.00 |
| KIM-1 | 318268.00 | 266037.00 | 386459.00 | 305734.00 |
|  | 184959.00 | 287357.00 | 406074.00 | 303113.00 |
|  | 177088.00 | 295826.00 | 394135.00 | 282011.00 |
|  | 325124.00 | 300380.00 | 412290.00 | 282990.00 |
| KIM-1/Bactin | 1.04 | 0.81 | 1.09 | 0.92 |
|  | 0.57 | 0.83 | 1.10 | 0.86 |
|  | 0.60 | 0.93 | 1.22 | 1.07 |
|  | 0.97 | 0.90 | 1.26 | 0.98 |

Supplementary B7. Original Integrated Density of β-actin and KIM-1 for Fig.4G

| Cis-10uM | - | + | + | + | + |
| --- | --- | --- | --- | --- | --- |
| SAC(uM) | - | - | 10 | 30 | 100 |
| Bactin | 228926.00 | 224327.00 | 300898.00 | 237982.00 | 208651.00 |
|  | 238580.00 | 237830.00 | 270419.00 | 242611.00 | 211168.00 |
|  | 266710.00 | 258950.00 | 299678.00 | 241913.00 | 220372.00 |
|  | 262424.00 | 274847.00 | 273904.00 | 261688.00 | 204992.00 |
|  | 305293.00 | 277129.00 | 266574.00 | 257621.00 | 220864.00 |
|  | 291472.00 | 257811.00 | 259417.00 | 255114.00 | 214297.00 |
| KIM-1 | 207537.00 | 346896.00 | 348817.00 | 237516.00 | 130771.00 |
|  | 214417.00 | 433719.00 | 341481.00 | 242052.00 | 138887.00 |
|  | 264183.00 | 411566.00 | 372932.00 | 257431.00 | 102998.00 |
|  | 229494.00 | 340376.00 | 360841.00 | 271810.00 | 132798.00 |
|  | 222626.00 | 396260.00 | 343351.00 | 249995.00 | 130791.00 |
|  | 228196.00 | 291729.00 | 358054.00 | 242665.00 | 109421.00 |
| KIM-1/Bactin | 0.91 | 1.55 | 1.16 | 1.00 | 0.63 |
|  | 0.90 | 1.82 | 1.26 | 1.00 | 0.66 |
|  | 0.99 | 1.59 | 1.24 | 1.06 | 0.47 |
|  | 0.87 | 1.24 | 1.32 | 1.04 | 0.65 |
|  | 0.73 | 1.43 | 1.29 | 0.97 | 0.59 |
|  | 0.78 | 1.13 | 1.38 | 0.95 | 0.51 |

Supplementary table8. Original Integrated Density of β-actin and NGAL for Fig4H

| Cis-10uM | - | + | + | + | + |
| --- | --- | --- | --- | --- | --- |
| SAC(uM) | - | - | 10 | 30 | 100 |
| Bactin | 228926.00 | 224327.00 | 300898.00 | 237982.00 | 208651.00 |
|  | 238580.00 | 237830.00 | 270419.00 | 242611.00 | 211168.00 |
|  | 266710.00 | 258950.00 | 299678.00 | 241913.00 | 220372.00 |
|  | 262424.00 | 274847.00 | 273904.00 | 261688.00 | 204992.00 |
|  | 305293.00 | 277129.00 | 266574.00 | 257621.00 | 220864.00 |
|  | 291472.00 | 257811.00 | 259417.00 | 255114.00 | 214297.00 |
| NGAL | 233514.00 | 356999.00 | 346505.00 | 313083.00 | 169572.00 |
|  | 219650.00 | 324633.00 | 358353.00 | 331645.00 | 192747.00 |
|  | 216798.00 | 331427.00 | 334275.00 | 298277.00 | 205777.00 |
|  | 223403.00 | 319711.00 | 380274.00 | 248652.00 | 205707.00 |
|  | 260485.00 | 339040.00 | 374181.00 | 255969.00 | 191102.00 |
|  | 218753.00 | 310063.00 | 358760.00 | 269057.00 | 199331.00 |
| NGAL/Bactin | 1.02 | 1.59 | 1.15 | 1.32 | 0.81 |
|  | 0.92 | 1.36 | 1.33 | 1.37 | 0.91 |
|  | 0.81 | 1.28 | 1.12 | 1.23 | 0.93 |
|  | 0.85 | 1.16 | 1.39 | 0.95 | 1.00 |
|  | 0.85 | 1.22 | 1.40 | 0.99 | 0.87 |
|  | 0.75 | 1.20 | 1.38 | 1.05 | 0.93 |

Supplementary table9. Original Integrated Density of β-actin and FBP1 for Fig.J

| Cis 10uM | - | + | + | + | + |
| --- | --- | --- | --- | --- | --- |
| SAC (uM) | - | - | 10.00 | 30.00 | 100.00 |
| Bactin | 330614.00 | 270539.00 | 315416.00 | 333934.00 | 310938.00 |
|  | 306140.00 | 327846.00 | 270212.00 | 272816.00 | 334599.00 |
|  | 285348.00 | 273135.00 | 353228.00 | 298793.00 | 272381.00 |
|  | 312616.00 | 329180.00 | 308604.00 | 293039.00 | 323133.00 |
|  | 329303.00 | 335027.00 | 291619.00 | 312159.00 | 321217.00 |
|  | 305510.00 | 296960.00 | 310399.00 | 307094.00 | 307893.00 |
| FBP1 | 341280.00 | 262232.00 | 298440.00 | 448674.00 | 352037.00 |
|  | 374893.00 | 272703.00 | 294428.00 | 407698.00 | 387655.00 |
|  | 374713.00 | 291358.00 | 284896.00 | 350037.00 | 427166.00 |
|  | 401401.00 | 328198.00 | 305488.00 | 422592.00 | 369758.00 |
|  | 335182.00 | 319875.00 | 302400.00 | 477433.00 | 382050.00 |
|  | 349007.00 | 267920.00 | 278347.00 | 389922.00 | 295273.00 |
| FBP1/Bactin | 1.03 | 0.97 | 0.95 | 1.34 | 1.13 |
|  | 1.22 | 0.83 | 1.09 | 1.49 | 1.16 |
|  | 1.31 | 1.07 | 0.81 | 1.17 | 1.57 |
|  | 1.28 | 1.00 | 0.99 | 1.44 | 1.14 |
|  | 1.02 | 0.95 | 1.04 | 1.53 | 1.19 |
|  | 1.14 | 0.90 | 0.90 | 1.27 | 0.96 |

Supplementary table10. Original Integrated Density of β-actin and G6PC for Fig.4K

| Cis-10uM | - | + | + | + | + |
| --- | --- | --- | --- | --- | --- |
| SAC(uM) | - | - | 10.00 | 30.00 | 100.00 |
| Bactin | 330614.00 | 270539.00 | 315416.00 | 333934.00 | 310938.00 |
|  | 306140.00 | 327846.00 | 270212.00 | 272816.00 | 334599.00 |
|  | 285348.00 | 273135.00 | 353228.00 | 298793.00 | 272381.00 |
|  | 312616.00 | 329180.00 | 308604.00 | 293039.00 | 323133.00 |
|  | 329303.00 | 335027.00 | 291619.00 | 312159.00 | 321217.00 |
|  | 305510.00 | 296960.00 | 310399.00 | 307094.00 | 307893.00 |
| G6PC | 368801 | 535738 | 439177 | 469501 | 373373 |
|  | 374393 | 565637 | 374259 | 496881 | 419069 |
|  | 536240 | 489323 | 420404 | 528910 | 449996 |
|  | 437487 | 371130 | 443933 | 501486 | 426464 |
|  | 425025 | 409578 | 469069 | 435529 | 411101 |
|  | 522931 | 464245 | 484338 | 516652 | 448227 |
| G6PC/Bactin | 1.12 | 1.98 | 1.39 | 1.41 | 1.20 |
|  | 1.22 | 1.73 | 1.39 | 1.82 | 1.25 |
|  | 1.88 | 1.79 | 1.19 | 1.77 | 1.65 |
|  | 1.40 | 1.13 | 1.44 | 1.71 | 1.32 |
|  | 1.29 | 1.22 | 1.61 | 1.40 | 1.28 |
|  | 1.71 | 1.56 | 1.56 | 1.68 | 1.46 |

Supplementary table11. Original Integrated Density of β-actin and PCK1 for Fig4L

| Cis-10uM | - | + | + | + | + |
| --- | --- | --- | --- | --- | --- |
| SAC(uM) | - | - | 10.00 | 30.00 | 100.00 |
| Bactin | 330614.00 | 270539.00 | 315416.00 | 333934.00 | 310938.00 |
|  | 306140.00 | 327846.00 | 270212.00 | 272816.00 | 334599.00 |
|  | 285348.00 | 273135.00 | 353228.00 | 298793.00 | 272381.00 |
|  | 312616.00 | 329180.00 | 308604.00 | 293039.00 | 323133.00 |
|  | 329303.00 | 335027.00 | 291619.00 | 312159.00 | 321217.00 |
|  | 305510.00 | 296960.00 | 310399.00 | 307094.00 | 307893.00 |
| PCK1 | 115105.00 | 118911.00 | 132482.00 | 116433.00 | 156887.00 |
|  | 130775.00 | 138575.00 | 132224.00 | 143833.00 | 153642.00 |
|  | 119581.00 | 133333.00 | 152297.00 | 130687.00 | 156695.00 |
|  | 147618.00 | 122344.00 | 146602.00 | 128956.00 | 142908.00 |
|  | 147937.00 | 145111.00 | 130091.00 | 155187.00 | 160350.00 |
|  | 133666.00 | 144826.00 | 111781.00 | 156735.00 | 155620.00 |
| PCK1/Bactin | 0.35 | 0.44 | 0.42 | 0.35 | 0.50 |
|  | 0.43 | 0.42 | 0.49 | 0.53 | 0.46 |
|  | 0.42 | 0.49 | 0.43 | 0.44 | 0.58 |
|  | 0.47 | 0.37 | 0.48 | 0.44 | 0.44 |
|  | 0.45 | 0.43 | 0.45 | 0.50 | 0.50 |
|  | 0.44 | 0.49 | 0.36 | 0.51 | 0.51 |

Supplementary table12. Original Integrated Density of β-actin and FBP1 for Fig6K.

| Cis | - | + | + | + |
| --- | --- | --- | --- | --- |
| SAC | - | - | + | + |
| siFBP1 | - | - | - | - |
| Bactin | 451330.00 | 377807.00 | 387465.00 | 424437.00 |
|  | 393651.00 | 456368.00 | 406662.00 | 416429.00 |
|  | 410538.00 | 409342.00 | 407324.00 | 441009.00 |
|  | 363566.00 | 357757.00 | 400144.00 | 502157.00 |
|  | 392860.00 | 402519.00 | 396235.00 | 503226.00 |
|  | 388567.00 | 415493.00 | 414135.00 | 441884.00 |
| FBP1 | 202711.00 | 216611.00 | 225847.00 | 164848.00 |
|  | 211502.00 | 199009.00 | 201152.00 | 145036.00 |
|  | 259742.00 | 140972.00 | 233030.00 | 149919.00 |
|  | 253363.00 | 178662.00 | 229887.00 | 167904.00 |
|  | 236011.00 | 184370.00 | 272040.00 | 208290.00 |
|  | 264921.00 | 188641.00 | 238798.00 | 186643.00 |
| FBP1/Bactin | 0.45 | 0.57 | 0.58 | 0.39 |
|  | 0.54 | 0.44 | 0.49 | 0.35 |
|  | 0.63 | 0.34 | 0.57 | 0.34 |
|  | 0.70 | 0.50 | 0.57 | 0.33 |
|  | 0.60 | 0.46 | 0.69 | 0.41 |
|  | 0.68 | 0.45 | 0.58 | 0.42 |

Supplementary table13. Original Integrated Density of β-actin and NGAL for Fig6L.

| Cis | - | + | + | + |
| --- | --- | --- | --- | --- |
| SAC | - | - | + | + |
| siFBP1 | - | - | - | - |
| Bactin | 451330.00 | 377807.00 | 387465.00 | 424437.00 |
|  | 393651.00 | 456368.00 | 406662.00 | 416429.00 |
|  | 410538.00 | 409342.00 | 407324.00 | 441009.00 |
|  | 363566.00 | 357757.00 | 400144.00 | 502157.00 |
|  | 392860.00 | 402519.00 | 396235.00 | 503226.00 |
|  | 388567.00 | 415493.00 | 414135.00 | 441884.00 |
| NGAL | 257693.00 | 332306.00 | 313973.00 | 444089.00 |
|  | 200763.00 | 454759.00 | 283151.00 | 523992.00 |
|  | 164469.00 | 461054.00 | 272191.00 | 510650.00 |
|  | 252169.00 | 379935.00 | 258939.00 | 475651.00 |
|  | 222246.00 | 455811.00 | 331544.00 | 453026.00 |
|  | 211228.00 | 377220.00 | 402852.00 | 493162.00 |
| NGAL/Bactin | 0.57 | 0.88 | 0.81 | 1.05 |
|  | 0.51 | 1.00 | 0.70 | 1.26 |
|  | 0.40 | 1.13 | 0.67 | 1.16 |
|  | 0.69 | 1.06 | 0.65 | 0.95 |
|  | 0.57 | 1.13 | 0.84 | 0.90 |
|  | 0.54 | 0.91 | 0.97 | 1.12 |
